# Long-term Effects of Calcium β-Hydroxy-β-Methylbutyrate and Vitamin D^3^ Supplementation on Muscular Function in Older Adults With and Without Resistance Training: A Randomized, Double-blind, Controlled Study

**DOI:** 10.1093/gerona/glaa218

**Published:** 2020-08-28

**Authors:** John A Rathmacher, Lisa M Pitchford, Paul Khoo, Hector Angus, James Lang, Kristin Lowry, Carol Ruby, Alex C Krajek, John C Fuller, Rick L Sharp

**Affiliations:** 1 MTI BioTech, Inc., Iowa State University Research Park, Ames; 2 Department of Animal Science, Iowa State University, Ames; 3 Department of Kinesiology, Iowa State University, Ames; 4 Department of Physical Therapy, Des Moines University; 5 Metabolic Technologies, LLC, Missoula, MT

**Keywords:** β-Hydroxy-β-methylbutyrate, Aging, Functionality, Muscle strength, Vitamin D

## Abstract

The primary aim of this study was to determine whether supplementation with calcium β-hydroxy-β-methylbutyrate (HMB) and vitamin D_3_ (D) would enhance muscle function and strength in older adults. Older adults over 60 years of age with insufficient circulating 25-hydroxy-vitamin D (25OH-D) levels were enrolled in a double-blinded controlled 12-month study. Study participants were randomly assigned to treatments consisting of: (a) Control + no exercise, (b) HMB+D + no exercise, (c) Control + exercise, and (d) HMB+D + exercise. The study evaluated 117 participants via multiple measurements over the 12 months that included body composition, strength, functionality, and questionnaires. HMB+D had a significant benefit on lean body mass within the nonexercise group at 6 months (0.44 ± 0.27 kg, HMB+D vs −0.33 ± 0.28 kg, control, *p* < .05). In nonexercisers, improvement in knee extension peak torque (60°/s) was significantly greater in HMB+D-supplemented participants than in the nonsupplemented group (*p =* .04) at 3 months, 10.9 ± 5.7 Nm and −5.2 ± 5.9 Nm, respectively. A composite functional index, integrating changes in handgrip, Get Up, and Get Up and Go measurements, was developed. HMB+D + no exercise resulted in significant increases in the functional index compared with those observed in the control + no exercise group at 3 (*p =* .03), 6 (*p =* .04), and 12 months (*p =* .04). Supplementation with HMB+D did not further improve the functional index within the exercising group. This study demonstrated the potential of HMB and vitamin D_3_ supplementation to enhance muscle strength and physical functionality in older adults, even in individuals not engaged in an exercise training program.

Lean body mass (LBM) decreases at a rate of about 8% per decade after the age of 40 (1,2) and accelerates to about 15% per decade after the age of 70 (2). Decreasing LBM typically reflects a loss of muscle mass and is accompanied by reduced muscular strength and physical function. These losses have serious, wide-ranging implications for older adults. LBM and strength are inversely associated with loss of independence, fall risk (3), morbidity, and mortality (4,5). Thus, attenuating the age-related losses of muscle mass and function has great potential to improve health and quality of life.

Several strategies have been proposed to slow age-related muscle loss, but to date, only resistance training, alone or in combination with nutritional interventions, has been consistently shown to be effective (6,7). However, nutritional interventions alone are generally only effective in cases of restricted food intake or overt malnutrition (8). Insufficient protein intake (less than the recommended daily allowance [RDA] of 0.8 g per kg per day) is associated with reduced LBM and physical performance (9,10). Although protein insufficiency affects relatively few older adults (~10%) (11,12), increasing protein intake above the RDA increases muscle mass but does not improve muscle strength or global physical functioning (13,14). Similarly, pharmacological interventions, primarily using anabolic agents, have been less convincing with some studies showing beneficial (6,15) and others showing adverse outcomes (16). Additionally, the use of anabolic hormones has been associated with significant morbidities, limiting the utility of these agents in the general population.

Our group (17,18) and several others (19–22) have shown that HMB (β-hydroxy-β-methyl-butyrate), a metabolite of the essential amino acid, leucine, has beneficial effects on muscle mass, muscle strength, muscle function, and protein kinetics in older and young adults (23–25). In two randomized double-blind studies, Flakoll et al. (18) found that daily supplementation with calcium HMB and two amino acids, arginine and lysine, (HMB/Arg/Lys) for 12 weeks significantly increased muscle strength and improved functionality and tended to enhance the gain of muscle mass; these effects were attributed to increased whole-body protein synthesis. In a subsequent year-long study by Baier et al. (17), daily supplementation with HMB/Arg/Lys significantly improved LBM in supplemented older adults but showed no improvements in muscle strength or function. A retrospective analysis of the same data (26) revealed that the benefit on muscle strength was highly dependent on the circulating levels of vitamin D; the participants who had sufficient vitamin D levels (25-hydroxy-vitamin D ≥ 30 ng/mL, 25OH-D) showed significant improvements in muscle strength with HMB/Arg/Lys supplementation, whereas those with insufficient vitamin D levels did not. These findings suggest that adequate vitamin D levels may be necessary to achieve optimal benefits with HMB. Vitamin D insufficiency is highly prevalent (50%–75%) in older adults, despite efforts to encourage screening and replacement therapy (27,28), and vitamin D insufficiency is now recognized as an independent risk factor for accelerated muscle loss, poor physical performance, and falls (27). Vitamin D supplementation can quickly and effectively increase 25OH-D to sufficient levels (29). Based on these observations, we hypothesized that co-supplementation with calcium HMB and vitamin D_3_ would enhance muscle strength and function and lead to decreased falls and improved quality of life for older adults. Furthermore, we hypothesized that the addition of a modest resistance exercise regimen to these supplements will lead to even greater benefits in the combined intervention group.

The primary aim of this study was to determine whether supplementation with calcium HMB in combination with vitamin D_3_ aimed at achieving sufficient circulating levels of vitamin D would enhance muscle function and strength in older adults. Given the demonstrated benefit of resistance exercise training on these factors in older adults, we sought to evaluate the benefit of calcium HMB and vitamin D_3_ supplementation both with and without concurrent moderate exercise training.

## Method

This 12-month clinical trial employed a randomized, double-blind, placebo-controlled 2 × 2 factorial design. The experiment was double-blind with respect to calcium HMB plus vitamin D_3_ (HMB+D) and control supplementation. Participants were stratified by sex and assigned to one of the four treatment arms using computer-generated random numbers. The treatment arms consisted of: (a) Control + no exercise, (b) HMB+D + no exercise, (c) Control + exercise, and (d) HMB+D + exercise. The clinical trial consisted of multiple measurements over the 12 months. Assessments were conducted at a single location (Nutrition and Wellness Research Center, Ames, IA) and, except for dual energy x-ray absorptiometry (DXA), were performed at baseline and again at 3, 6, 9, and 12 months. The assessments were conducted in the following order: blood pressure and heart rate, blood sampling, body composition, and function testing. The questionnaires were completed as time allowed or at the end of testing. The participants were given a small meal at the end of testing. The assessors were blinded to the nutritional treatments. This study was registered at ClinicalTrials.gov (NCT02043171) and was approved by the Iowa State University Institutional Review Board (IRB00000473, ID:13-573). The National Institute on Aging appointed a three-member Data and Safety Monitoring Board (DSMB) and a medical monitor; the DSMB met on five occasions during the conduct of the study and received progress and safety updates. The administration of calcium HMB and vitamin D_3_ was conducted under Food and Drug Administration (FDA) Investigational New Drug (IND) 109079 according to 21 CFR 312.

### Participants

Men and women ≥ 60 years of age with insufficient, but not clinically deficient 25OH-D levels (baseline concentration between 15 and 30 ng/mL) were recruited for this study. Volunteers were solicited from a willing recruitment list, electronic mailings, United States Postal Service (USPS) mailings, and flyers for the study. Participants had a starting body mass index (BMI) of < 40 kg/m^2^, were free of liver and kidney diseases or other serious medical illnesses, had no evidence of uncontrolled hypertension, did not have osteoporosis or a bone density T-score < −2.0 or chronic diseases affecting calcium or bone metabolism, had no history of blood clots and/or the use of blood thinning medications, were able and willing to participate in 3-day-a-week monitored resistance-training program, had no major surgery in the previous 6 weeks, and did not have any restrictions placed on physical exercise by their primary care physician. If at follow-up, a participant had a 25OH-D < 12 ng/mL or T-score < −2.5, the participant was referred to a physician and was dropped from the study.

### Nutritional Supplements

Supplements consisted of either a placebo (calcium lactate) in the control group or the combination of calcium HMB (3.0 g/day) plus vitamin D_3_ (2,000 IU/day) in the supplemented (HMB+D) group. This HMB dosing strategy (3 g/d, split into two doses) has been utilized in the majority of previous studies examining the effects of HMB on body composition and physical and function performance in older adults (19,20). Vitamin D doses ranging from 800 to 2000 IU per day have been recommended to achieve a minimum serum 25OH-D of 30 ng/mL at 3 months (29). The vitamin D_3_ dosing strategy (2,000 IU/d, split into two doses) was utilized in this study to rapidly increase circulating levels of 25OH-D to be within the sufficient range (30–100 ng/mL) where HMB has been previously shown to be efficacious for muscle strength improvements (26). Both nutritional supplements were provided in capsules of equal size, color, and taste and were produced in a current Good Manufacturing Practice (cGMP) facility and obtained through TSI Innovative Products Division (Missoula, MT). The purity of calcium HMB used in the capsules was determined by the manufacturer using high-pressure liquid chromatography (HPLC) to be greater than 98%. The calcium HMB and vitamin D_3_ concentrations of the capsules were verified throughout the study (Heartland Assays, Ames, IA). Capsules were consumed twice daily with the morning and evening meals. Both supplements contained equal amounts of calcium (102 mg), phosphorus (26 mg), and potassium (49 mg). Prior to enrollment in the study, participants were instructed to discontinue any supplements containing HMB or vitamin D, but a multivitamin was allowed; this was maintained throughout the study period.

Exercise

Participants assigned to the moderate resistance exercise training program performed approximately 60 minutes of supervised resistance training three times per week (30) in two dedicated exercise studios located in Ames, IA and Des Moines, IA. Participants were permitted to exercise outside of the studios with bands when traveling or confined to home. The resistance exercise program consisted of biceps curls, triceps extensions, chair squats, calf raises, ankle dorsiflexion, shoulder front raises and lateral raises, latissimus dorsi pull-down, chest press, seated row, knee flexion and extension, and hip flexion. Participants completed three sets of each exercise, including two sets up to 15 repetitions and a final set of up to 20 repetitions. Initially, Thera-Band (Duluth, GA) stretch cords were used for exercise resistance. Once a participant was able to complete 20 repetitions with good form, the resistance was increased by moving to the next color of resistance band. Hops or small jumps were performed between exercises (5 hops after each set, increasing by 5 hops per week until 25 hops were achieved). Resistance band exercise has been shown to safely increase strength and functionality when used in an older adult population (31,32). However, once participants increased their muscle strength beyond use of Thera-Bands, they were transitioned to resistance training on machines to perform the same exercises.

The resistance exercise machines utilized were commercially available cable-pulley and plate loaded equipment pieces. While the repetition range and number of exercises were similar to the Thera-Band phase, transition to use of machine equipment allowed participants to achieve larger resistance loads. The participants’ rest time between sets and weekly exercise session number were kept similar to the protocol for the Thera-Band phase. Progression of load for machine exercises followed the guidelines set by the American College of Sports Medicine, whereby load was increased by 2%–10% when the participant felt they could achieve 1–2 more repetitions over the 20th repetition on the third set (33). Increasing load for the resistance exercise machines was an addition of weighted plates to a load stack being moved by the participants (33). The same resistance exercise session supervisors were also utilized to minimize variability with resistance prescription and progression. The modifications between equipment phases were augmentation from chair squats to a machine sled leg press, standing unilateral knee flexion with Thera-Bands to a seated bilateral knee flexion movement, and overhead unilateral triceps extensions to a bilateral triceps extension using a pulldown movement. The nonexercise groups were instructed not to perform resistance exercise during the study period.

### Measurements

#### Body weight and composition

Body weight was measured without shoes following an overnight fast. DXA (Hologic Discovery v.12.3) was used to assess regional body composition (lean and fat mass) and bone density data at 0, 6, and 12 months only. Bioelectrical impedance analysis (BIA; BIA-101S, RJL Systems, Clinton Township, MI) and air displacement plethysmography (ADP; BOD POD, LMI, Concord CA) (34) were used to measure body composition at all timepoints. BIA data were analyzed using the Fluid & Nutrition Analysis Software, version 3.1b (RJL Systems) (35) and ADP calculations were performed using the Siri equation (36). Body composition measurements were conducted by the same researcher over the 12-month period. Previous publications have shown a high correlation between ADP, BIA, and DXA measurements (37).

#### Muscle strength

Muscle strength was assessed via isokinetic dynamometry. Bilateral knee and elbow extension/flexion peak torque were measured at multiple speeds (knee: 60, 90, and 180°/s; elbow: 60 and 120°/s) using the BIODEX Isokinetic Dynamometer (System 3 Quickset, Shirley, NY). Peak torque generation for each movement and speed was also analyzed independently. Strength measurements were conducted by the same researcher over the 12-month period. Additionally, a total lower composite extremity strength index was calculated to examine the effect of the intervention on overall lower extremity muscle function. Lower Extremity Strength Index = (left leg extension peak torque at 60°/s + 90°/s + 180°/s) + (right leg extension peak torque at 60°/s + 90°/s + 180°/s) + (left leg flexion peak torque at 60°/s + 90°/s + 180°/s) + (right leg flexion peak torque at 60°/s + 90°/s + 180°/s).

#### Physical function

The “Timed Up-and-Go” and “Get-up” tests were used to assess physical function. The “Up-and-Go” test requires the participant to, starting from a seated position, stand, walk forward 3 m, turn around, walk back to the chair, and sit down as quickly as possible without running (38); three “Up-and-Go” trials were performed, and the average time was recorded. The “Get-up” test (30 second sit to stand) requires the participant to stand up from a seated position as many times as possible within 30 seconds (38). Handgrip strength was measured using a handgrip dynamometer (Lafayette Instrument Co., Lafayette, IN); three trials were completed per side, the average for each side was recorded, and the sum of left and right handgrip was used for analysis. A composite functional index was developed to assess additive improvement across multiple muscle groups and has transitional properties that capture changing improvement in functional status. The index of changes (Composite Functional Index) was calculated as the sum of fractional changes in all functionality measures [left handgrip + right handgrip + Get Up + (−Get Up and Go)].

#### Dietary assessment

Food recalls (3 days) were used to estimate vitamin D and nutrient intake at all timepoints. Records were analyzed using the Food Processor (ESHA Research, Salem OR).

#### Blood sampling

Blood and urine samples collected after an overnight fast were analyzed by LabCorp (Urbandale, IA) for basic chemistry profile, complete blood count with differential, and urinalysis at screening and at all timepoints. In addition, blood levels of bone alkaline phosphatase, 25OH-D, and parathyroid hormone (PTH) were analyzed by Heartland Assays (Ames, IA) using the Liaison XL automated chemiluminescence analyzer.

#### Questionnaires

A health form questionnaire, quality of life questionnaire (SF-36 Health Survey) (39), and Circumplex Affect questionnaire (40) were completed by participants at each visit. Each participant also maintained a falls calendar.

#### Compliance

Compliance to the supplement protocol was monitored using participant logs, capsule counts, and by measuring serum 25OH-D concentrations.

#### Statistics

The primary outcome of this study was the improvement in muscle function and strength in an older adult population over 12 months. We hypothesized that combined supplementation with calcium HMB and vitamin D_3_ would also lead to decreased falls and to improved quality of life for older adults. We further hypothesized that the addition of a modest resistance exercise regimen to these supplements would enhance the synergistic effects of calcium HMB and vitamin D_3_. A priori power analysis (G-Power, v3.0, Universität Kiel, Germany) was completed based on knee strength data and vitamin D status from a retrospective data analysis from the study by Baier et al. (17). For the power analysis calculation, a 33.9 Nm increase in total leg strength was anticipated for the treatment group during the 12-month study, whereas a 10.0 Nm change in total leg strength was expected to occur in the control group. The power analysis was based on an *F*-test (Analysis of Variance [ANOVA]: Repeated measure with 5 time observations and 4 treatment groups) with an α-error probability of 0.05, and power of 0.8; it was estimated that 20 participants per treatment with adequate vitamin D status would be needed to detect significant changes in muscle strength. To assure adequate numbers of participants finished the entire protocol, we assumed a drop rate of 33% and planned to enroll 40 participants per treatment. Body composition, function, and strength data were analyzed using a SAS Proc Mixed model ANOVA (Version 9.4, SAS Institute Inc., Cary, NC) on the change at 3, 6, 9, and/or 12 months. The model included sex, treatment, exercise, and treatment by exercise interaction and included the starting value as the covariate. Only those participants completing 12 months of study were included in the per-protocol analysis. Participants who completed at least 6 months of the study (*n* = 129) were included in a modified intent-to-treat analysis (ITT) (see Supplementary Figures 10–12 and Supplementary Table 11). The ITT data were analyzed with the same statistical models as the per-protocol statistical analysis. Post hoc *t*-tests were performed where significant treatment or treatment by exercise interactions were observed. As the primary aim of this study was to evaluate the effect of HMB+D on muscle function and strength, preplanned contrasts were used to evaluate the effect of HMB+D versus control supplementation on LBM, strength, and functional tests within exercising and nonexercising groups and data were expressed as the Mean ± SE. Clinical laboratory data were analyzed using a SAS Proc Mixed repeated-measure ANOVA. The model included starting value, sex, treatment, exercise, time, treatment by exercise interaction, treatment by time interaction, exercise by time interaction, and treatment by exercise by time interaction and data were expressed as the Mean ± SD. Adverse event questionnaires were analyzed as categorical data; the main effect of treatment was determined using the Cochran–Mantel–Haenszel test. Statistical significance was defined as *p* < .05 for all tests. Effect sizes were calculated from adjusted means and SE using Cohen’s *d*.

## Results

A total of 591 older adults were screened for this study. Of these, 238 participants were enrolled. A total of 117 participants completed the study and were included in the per-protocol analysis (Supplementary Figure 1). Baseline participant characteristics and functional data are given in Table 1. There were no differences in capsule supplementation and exercise compliance between groups as illustrated in Supplementary Table 1. The average group capsule compliance based on capsule count was 96.0 ± 0.4% and the average exercise compliance between the two exercise groups was 83.3 ± 0.3% based on attended exercise sessions and reported home exercise sessions.

**Table 1.** Baseline Participant Characteristics*

	HMB+D (No EX)	Control (No EX)	HMB+D (EX)	Control (EX)
*N*	27	26	30	34
Sex (M/F)	15/12	18/8	16/14	22/12
Age (y)	71.0 ± 1.1	70.8 ± 1.1	67.2 ± 0.7	67.7 ± 0.7
Weight (kg)	87.1 ± 3.9	93.0 ± 2.9	86.9 ± 3.7	85.2 ± 3.0
BMI (kg/m^2^)	28.9 ± 1.0	31.8 ± 0.9	27.6 ± 0.8	28.3 ± 0.9
Lean Mass (kg)^†^	50.0 ± 2.2	53.7 ± 1.7	50.4 ± 2.2	51.6 ± 2.0
Body Fat (%)^†^	40.7 ± 0.9	40.1 ± 1.6	39.4 ± 1.2	37.9 ± 1.1
Functional data				
Get Up (reps)	16.9 ± 1.1	18.0 ± 1.0	17.7 ± 0.9	18.6 ± 0.9
Get Up & Go (s)	6.7 ± 0.2	7.1 ± 0.5	6.4 ± 0.2	6.1 ± 0.1
Grip strength (kg)	23.1 ± 1.9	26.3 ± 3.1	24.5 ± 2.0	26.7 ± 1.8

*Data are expressed as number (sex) or mean ± standard error of the mean. BMI = body mass index; HMB = calcium β-hydroxy-β-methylbutyrate; D = vitamin D_3_; EX = Exercise.

^†^Measured using dual x-ray absorptiometry.

### Vitamin D Status

**Figure 1.**
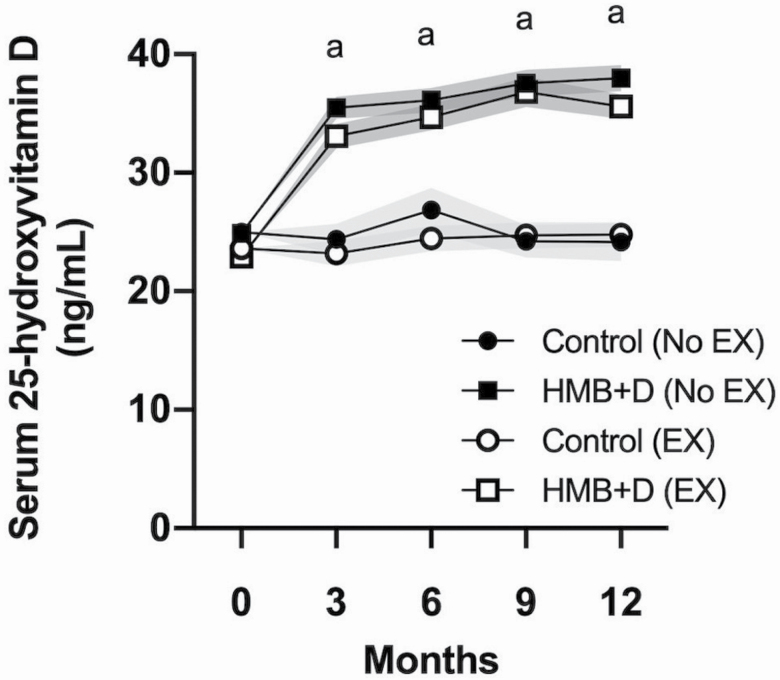
Serum 25-hydroxyvitamin D level. ^a^Main effect of HMB+D supplementation, *p* < .05. Data are expressed as mean ± SE (shaded area). HMB = calcium β-hydroxy-β-methylbutyrate; D = vitamin D_3_; EX = Exercise.

As designed, HMB+D supplementation resulted in significant increases in circulating total 25OH-D levels, which were maintained throughout the study period (Figure 1). There were no changes in circulating 25OH-D levels in either group that did not receive HMB+D supplementation, and there was no effect of exercise on 25OH-D levels. There were no treatment or exercise effects on serum levels of bone alkaline phosphatase or PTH (Supplementary Table 3). Reported macronutrient, vitamin D, and calcium intake were similar among groups and did not change over the course of the study (data not shown).

### Body Composition

**Figure 2.**
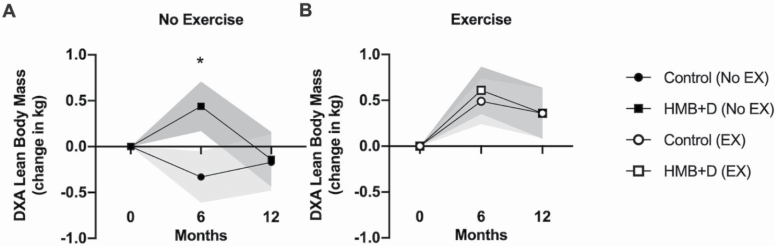
Changes in lean body mass as measured by dual x-ray absorptiometry. *Significant difference between HMB+D and control within group (no exercise or exercise); preplanned contrast, *p* < .05. Data are expressed as mean ± SE (shaded area). HMB = calcium β-hydroxy-β-methylbutyrate; D = vitamin D_3_; EX = Exercise.

HMB+D supplementation alone had a significant benefit on LBM within the nonexercise group at 6 months (0.44 ± 0.27 kg in HMB+D vs. −0.33 ± 0.28 kg in control, *p* < .05, *d =* 0.55), which was attributed to improvements in trunk lean mass (Trt main effect, *p* < .05, Supplementary Table 2); however, this benefit was not sustained through the 12-month time point (Figure 2A). The results from the BIA and Bod Pod analysis can be found in Supplementary Table 2.

### Functional Outcomes

**Figure 3.**
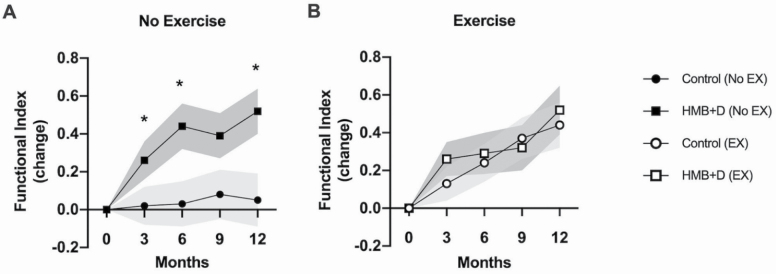
Changes in composite functional index (sum of fractional improvement in Get Up, Get Up and Go, and right and left handgrip strength). There was a significant treatment main effect (*p =* .03) of HMB+D supplementation at 3, 6, and 12 months. *Significant difference between HMB+D and control within group (no exercise or exercise); preplanned contrast, *p* < .05. Data are expressed as mean ± SE (shaded area). HMB = calcium β-hydroxy-β-methylbutyrate; D = vitamin D_3_; EX = Exercise.

As previously noted, a composite functional index was developed to assess additive improvements across multiple muscle groups [left handgrip + right handgrip + Get Up + (− Get Up and Go)]. The effect of HMB+D supplementation on the functional index was most prominent in the nonexercise group. HMB+D supplementation alone resulted in a larger increase in composite functional index than was observed in the control group at 3 months (*p =* .03, *d =* 0.58); even greater increases were observed at 6 months (*p =* .04, *d =* 0.70) and 12 months (*p =* .0*4*, *d =* 0.67), as shown in Figure 3A. Interestingly, supplementation with HMB+D did not further improve the functional index within the exercising group (Figure 3B).

Examination of each component of the functional index (Get Up, Get Up and Go, hand grip strength) in the exercising groups revealed similar patterns across the three components. The nonexercising nonsupplemented control group generally showed little to no improvement. However, improvements were observed amongst the supplemented HMB+D alone group and the exercise group, with or without HMB+D (Supplementary Figures 2–4).

### Strength Outcomes

**Figure 4.**
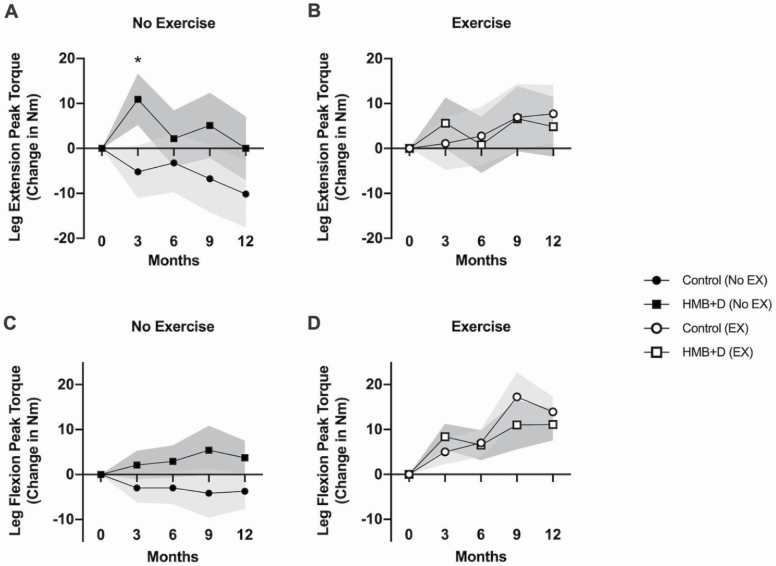
Changes in total (sum right and left legs) peak torque at 60°/s. (A) No exercise and (B) with exercise represent knee extension at 60°/s. There was a tendency (*p =* .056) for a main effect of HMB+D on leg extension peak torque at 3 months, and there was a significant difference (*) between the HMB+D and control groups within the no-exercise group (preplanned contrast, *p* < .05). (C) With no exercise and (D) with exercise represent knee flexion at 60°/s. There were main effects of exercise (*p* < .05) on leg flexion peak torque at 3, 6, 9, and 12 months. Data are expressed as mean ± SE (shaded area). HMB = calcium β-hydroxy-β-methylbutyrate; D = vitamin D_3_; EX = Exercise.

In nonexercisers, improvement in knee extension peak torque (60°/s) was significantly greater in HMB+D-supplemented participants than in the nonsupplemented group at 3 months (10.9 ± 5.7 vs. -5.2 ± 5.9 Nm, respectively, *p =* .04). Although the differences between groups at subsequent timepoints were not statistically significant, the control-supplemented participants continued to lose leg extension strength (−10.1 ± 7.4 Nm at 12 months), whereas strength was maintained at baseline levels in the HMB+D-supplemented participants (Figure 4A). There was no additional benefit of combining exercise and HMB+D supplementation on knee extension peak torque (Figure 4B). HMB+D supplementation did not significantly affect knee flexion peak torque. However, it is worth noting that among nonexercisers, peak torque decreased from baseline to 12 months in the nonsupplemented participants (−3.71 ± 3.91 Nm) as shown in Figure 4C. Exercise, either alone or in combination with HMB+D, showed similar improvements in knee flexion peak torque (main effect of exercise, *p* < .05, Figure 4D).

HMB+D supplementation tended to improve lower extremity strength index values among nonexercisers at 3 months (*p = .*10, *d* = 0.45). This tendency persisted at 9 and 12 months (*p =* .10 and .07, respectively). Among nonexercisers, HMB+D-supplemented participants maintained a similar improvement to that observed at 3 months (0.82 ± 0.29) throughout the year-long study while control participants remained near baseline values (0.04 ± 0.30, *d* = 0.51) (Supplementary Figure 7). Additional strength data (knee extension and flexion at 90°/s and 180°/s and elbow extension and flexion at 60°/s and 120°/s) are presented in Supplementary Figures 5–6 and 8–9.

### Falls

Neither HMB+D supplementation nor exercise influenced the number of falls recorded during the study period. Older adults supplemented with HMB+D averaged 0.75 ± 0.13 falls over the 12-month study period, and nonsupplemented participants had 0.51 ± 0.13 falls over the same period.

### Quality of Life

High activation as measured by the Circumplex Affect questionnaire increased significantly from baseline to 6, 9, and 12 months only in HMB+D-supplemented participants (Supplementary Table 9). No significant effects of treatment were observed for other Circumplex Affect categories or for SF-36 scores (Supplementary Table 10).

### Safety

No clinically significant effects of HMB+D supplementation were observed in blood or urine markers of hepatic or renal function, blood chemistry, blood lipids, or hematology (Supplementary Tables 4–7), nor were there any HMB+D related differences in adverse event frequency. Resting heart rate and blood pressure were also unaffected by HMB+D supplementation (Supplementary Table 8).

## Discussion

This study is the first long-term prospective investigation directly comparing calcium HMB and vitamin D_3_ supplementation in exercising and nonexercising healthy older adults. Combined supplementation with HMB and vitamin D_3_ for 12 months was safe and increased circulating levels of 25OH-D to within the sufficient range (30–100 ng/mL) previously shown to support a beneficial effect of HMB on lower body strength (26). The main finding of this study was that co-supplementation with calcium HMB and vitamin D_3_ to healthy older adults improved the composite functional strength index and that this benefit was independent of a moderate resistance exercise program similar to that commonly practiced by older populations on their own. In contrast, supplementation with HMB and vitamin D_3_ had minimal improvements on the same primary end point when healthy older individuals participated in an exercise program over a 1-year period. Interestingly, the functional improvements occurred with noticeable improvements in lean mass only at 6 months and on leg strength only at 3 months. These findings underscore the potent effects of supplementation with calcium HMB on improving functionality in nonexercising, healthy older adults with proven vitamin D sufficiency. Furthermore, these findings demonstrate that the effectiveness of HMB is independent of the additional amino acids often included in the nutritional supplement formulas previously shown to be efficacious in older adults (17,18). The study also highlights the independent potent effects of a moderate exercise program on the same primary end point of functionality in this population.

Skeletal muscle loss and decreased functionality are hallmarks of aging, and if left unattended can result in sarcopenia and loss of essential daily functions necessary for mobility and quality of life. It is well established that sarcopenia is a universal prelude for worsening of multiple chronic diseases and for the development of frailty (6). Assessment of functionality in this aging population can be quite complex and is one of the greatest challenges to healthcare professionals (41). The loss of functional status that leads to physical frailty is associated with adverse health outcomes, long-term institutionalization, and mortality (41). In this regard, we elected in the present study to utilize a functional composite index to represent the primary end point of estimating changes in strength over the 1-year period. This index incorporated several tests (Get Up test, the Get Up & Go test, and the handgrip strength test) frequently used to evaluate deficits in common daily function. Among the individual functional tests, the largest relative improvement was observed for the Get Up test, which evaluates a common critical function (getting up from a chair); its performance requires muscle strength, power, and balance. The observed functional improvements seen in the present study generally align with the functional improvements in nonexercising older adults previously observed with HMB supplementation, given alone (19,20) or in combination with two other amino acids, glutamine and arginine (22), with arginine and lysine for a shorter duration (3 months (18)) or with oral protein supplements (21). The findings also support the previous finding that supplementation with HMB in a similar group of older adults participating in an exercise program did not result in an added benefit on muscle strength over exercise alone (20). The benefits of HMB on muscle health have been largely attributed to enhanced muscle protein metabolism (17,18,25). These studies suggest that the effects of HMB on aging muscle may be mediated by shifting muscle protein balance to increase protein synthesis and blunt protein degradation (25), but as muscle protein metabolism was not measured in the present study, this mechanism cannot be confirmed in this study cohort.

There is extensive evidence that exercise training, including both aerobic and resistance exercise, results in improved skeletal muscle strength and mass and balance in older adults (6,7); unfortunately, a significant portion of older adults are either unable or unwilling to exercise regularly. In contrast, the evidence for nutritional interventions is at best modest, even when combined with exercise, in the presence or absence of sarcopenia (7). Interestingly, our data demonstrate that prolonged supplementation with both calcium HMB and vitamin D_3_ is crucial for the enhancement of muscle function in nonexercising older adults to the level achieved with exercise. Curiously, these improvements occurred without a sustained significant enhancement of muscle mass, which may be due to the nonspecificity of DXA as an accurate measurement of LBM; DXA is an indirect measure of muscle mass and is not predictive of functional muscle (42). In contrast, a more recent publication by Duchowny et al. showed that direct measurement of muscle mass assessed by the D_3_-creatine dilution method was tightly associated with changes in functional performance (42). Taken together, the lack of a generalized increase in muscle mass may also be suggestive of a potential neural involvement (43), the nature of which remains to be elucidated.

Exercise has many beneficial effects on brain health, improving cognitive function and decreasing incidence of depression and stress (44). As with previously observed improvements in emotional balance (45,46), supplementation with HMB+D resulted in significant improvements in the emotion of “High Activation” on the Circumplex Affect questionnaire. Hence, the findings in the current study could be related to the improvements in the functional composite index, representing an enhanced state of functional reserve. Such an increase in functional reserve would lessen the relative effort of daily activities (eg, climbing stairs, carrying groceries), which could result in feeling more energetic. These effects represent another potential cross talk between the improvements in muscle function and the brain, as those seen with exercise, with a consequent beneficial effect by reducing depression-like symptoms. This positive effect has been ascribed to the enhancement in muscular expression of the enzyme kynurenine aminotransferase (KAT), which converts neurotoxic KYN into neuroprotective kynurenic acid (KYNA) (44).

It is important to note that our study design did not allow us to distinguish between the independent effects of HMB and of vitamin D_3_. A previous retrospective study by Fuller et al. (26) had strongly suggested the existence of a synergistic effect of calcium HMB and vitamin D supplementation in older adults, as the benefits of HMB were blunted in individuals with proven vitamin D insufficiency (blood levels < 30 ng/mL). Based on the current data, we strongly believe that the positive long-term effects on functional composite index observed in the supplemented, but nonexercising older adults, are primarily attributed to the benefits of HMB that are fully realized in vitamin D sufficiency. This is also supported, albeit indirectly, by the lack of relationship between vitamin D levels and the improvements in our primary outcome, irrespective of whether they received or not received HMB+D supplementation. Although vitamin D supplementation has been shown to have beneficial effects on muscle strength in some populations, these findings are largely restricted to older, often institutionalized, adults with very low baseline vitamin D levels (<12 ng/mL) (47). A recent study by Shea et al. (48) evaluated the effects of 12 months of vitamin D_3_ supplementation or placebo on lower extremity power and function in 100 older community-dwelling adults ≥60 years with low serum 25OH-D (20.2 ± 6.7 ng/mL). Vitamin D_3_ supplementation raised participants’ 25OH-D similarly to that observed in the present study (32.5 ± 5.1 ng/mL), but lower extremity mass, strength, power, and function did not differ compared with placebo. Vaes et al. (49) evaluated a frailer population with more severe vitamin D insufficiency/deficiency (~15 ng/mL). While 6 months of supplementation with vitamin D_3_ successfully raised 25OH-D levels (~32 ng/mL), strength and performance were not improved in this population, either.

In summary, this study demonstrated the potential of long-term co-supplementation with calcium HMB and vitamin D_3_ to enhance physical functionality and muscle strength in older adults, even in individuals not engaged in a resistance exercise training program. While adequate nutrition and participation in resistance exercise training remain valuable for the maintenance of muscle strength, quality, and function, the combined supplementation with calcium HMB and vitamin D_3_ may provide a unique protective effect for the substantial population of older adults who are unable or unwilling to exercise. Although the results of the present study are difficult to generalize to a sarcopenic/presarcopenic or frail population, we hypothesize that the benefits of HMB+D could be even more valuable in these individuals given their lower baseline functional status. Furthermore, HMB has been previously shown to have substantial benefits on muscle health in various populations with established sarcopenia, chronic disease patients, and individuals with temporary or ongoing limitations to physical activity (18,21,50).

## Supplementary Material

glaa218_suppl_Supplementary_Figures_S1_S12_Tables_S1_S11
