# Supplementary material for: Long-term Effects of Calcium β-Hydroxy-β-Methylbutyrate and Vitamin D3 Supplementation on Muscular Function in Older Adults With and Without Resistance Training: A Randomized, Double-blind, Controlled Study
Source: J Gerontol A Biol Sci Med Sci. 2020 Aug 28;75(11):2089–97. doi: 10.1093/gerona/glaa218 (PMC7566440; doi:10.1093/gerona/glaa218)
Supplement: glaa218_suppl_Supplementary_Figures_S1_S12_Tables_S1_S11 [file glaa218_suppl_supplementary_figures_s1_s12_tables_s1_s11.docx]

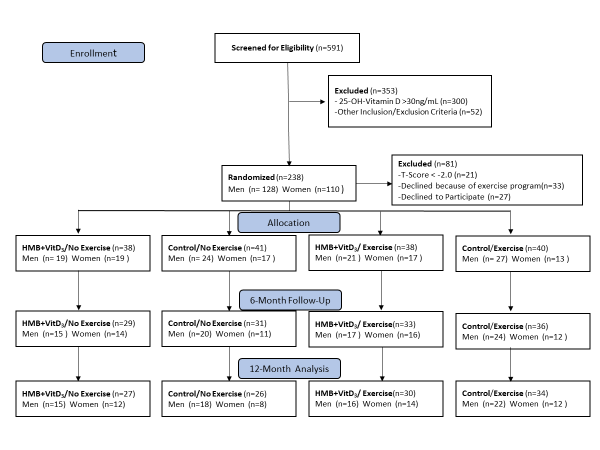
 **Supplemental Figure 1**. CONSORT Flow Diagram. HMB+D, calcium β-hydroxy-β-methylbutyrate + Vitamin D_3_.


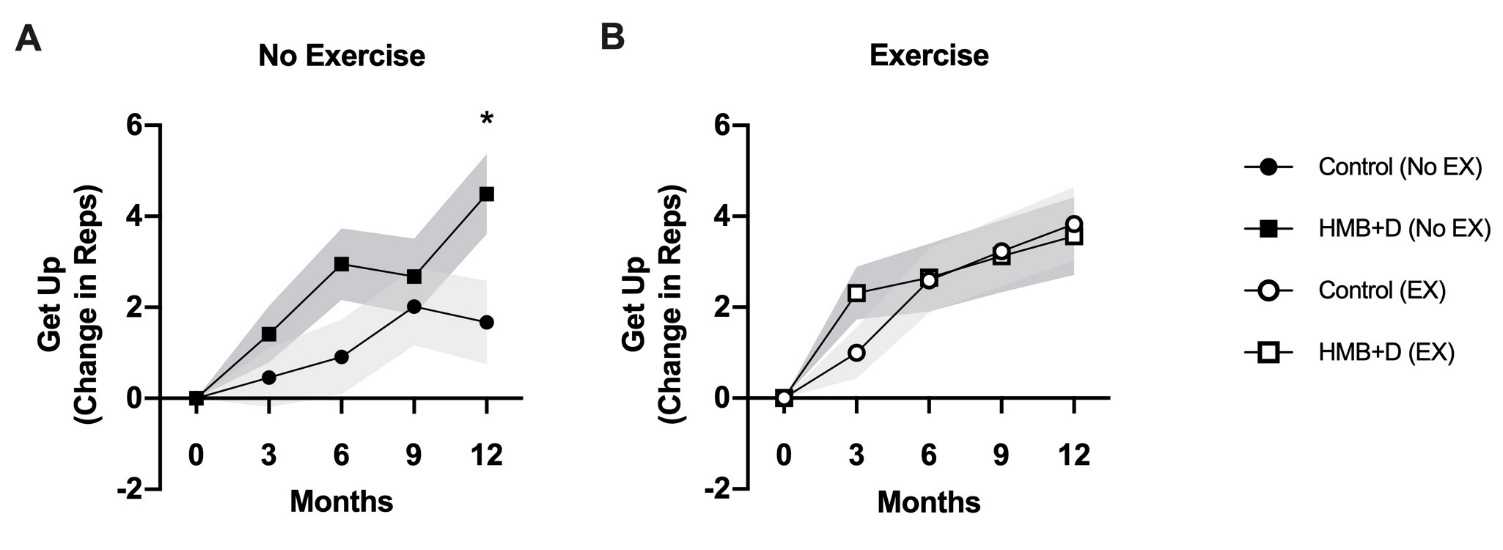
 **Supplemental Figure 2. Effect of HMB+D supplementation on change in Get Up test performance in non-exercising (A) and exercising (B) older adults.** There was a tendency for a main effect of HMB+D supplementation at 3 months (*p*=0.065) and a tendency for a supplementation*exercise interaction at 12 months (*p*=0.07). HMB+D supplementation alone tended to improve performance at 6 months (*p*=0.071, *d*=0.49) and significantly improved performance at 12 months (increase of 4.5 ± 0.9 reps in HMB+D vs. 1.7 ± 0.9 reps in control, *p*=0.03, *d*=0.61) in non-exercisers (A). Exercise resulted in a numerically similar improvement in Get Up test performance, but HMB+D supplementation did not further improve performance within the exercising group (B). *significant difference between HMB+D and control within group (no exercise or exercise); pre-planned contrast, *p*<0.05. Data are expressed as Mean ± SE (shaded area). HMB, calcium β-hydroxy-β-methylbutyrate. D, Vitamin D_3_. EX, Exercise.


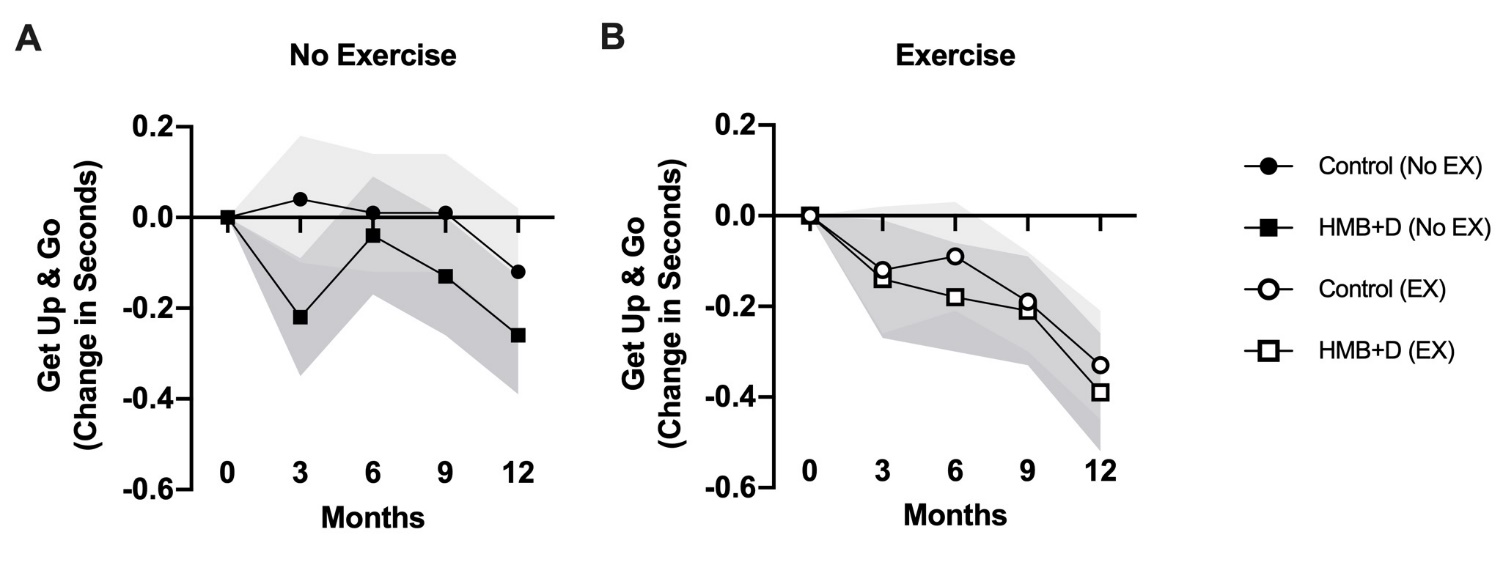


**Supplemental Figure 3. Effect of HMB+D supplementation on change in Get Up and Go test performance in non-exercising (A) and exercising (B) older adults.** No significant main effects or interactions were observed for Get Up and Go performance. Data are expressed as Mean ± SE (shaded area). HMB, calcium β-hydroxy-β-methylbutyrate. D, Vitamin D_3_. EX, Exercise.


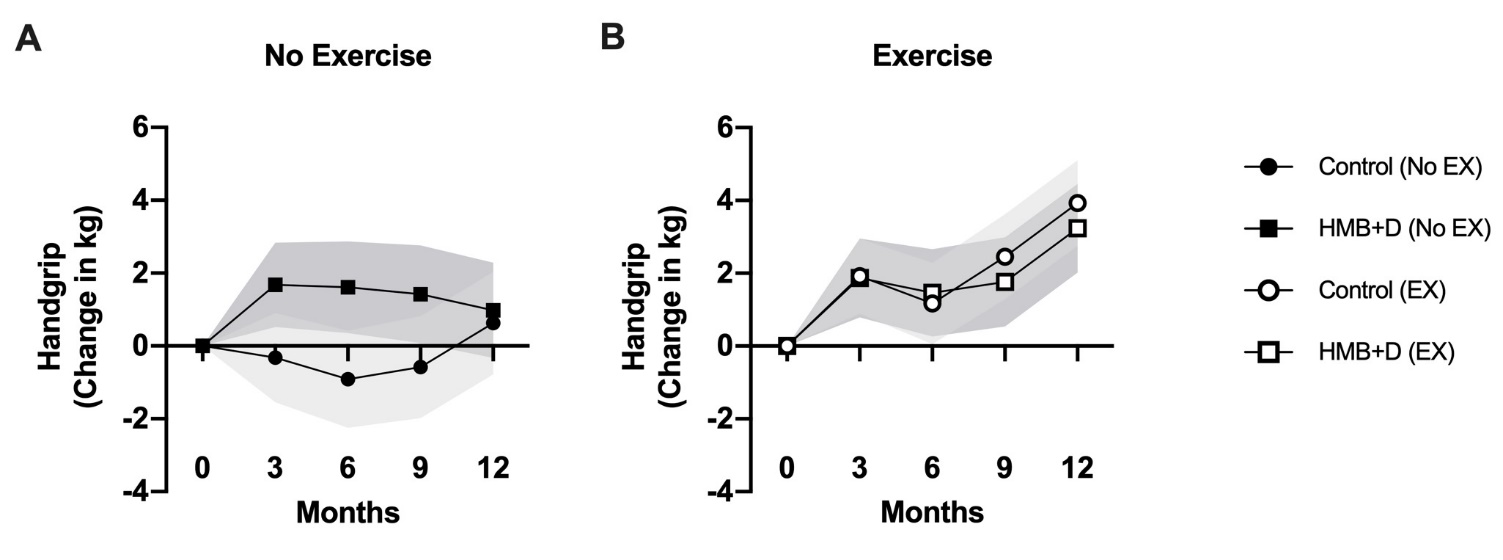


**Supplemental Figure 4. Effect of HMB+D supplementation on change in total (sum right+left) handgrip strength in non-exercising (A) and exercising (B) older adults.** There were no significant main or interaction effects for treatment on handgrip strength, but there was a main effect of exercise at 12 months (*p*=0.03). Though there were no significant differences between treatment groups, only the non-exercise control group experienced negative average changes in handgrip strength during the study period (3, 6, and 9 months). Data are expressed as Mean ± SE (shaded area). HMB, calcium β-hydroxy-β-methylbutyrate. D, Vitamin D_3_. EX, Exercise.


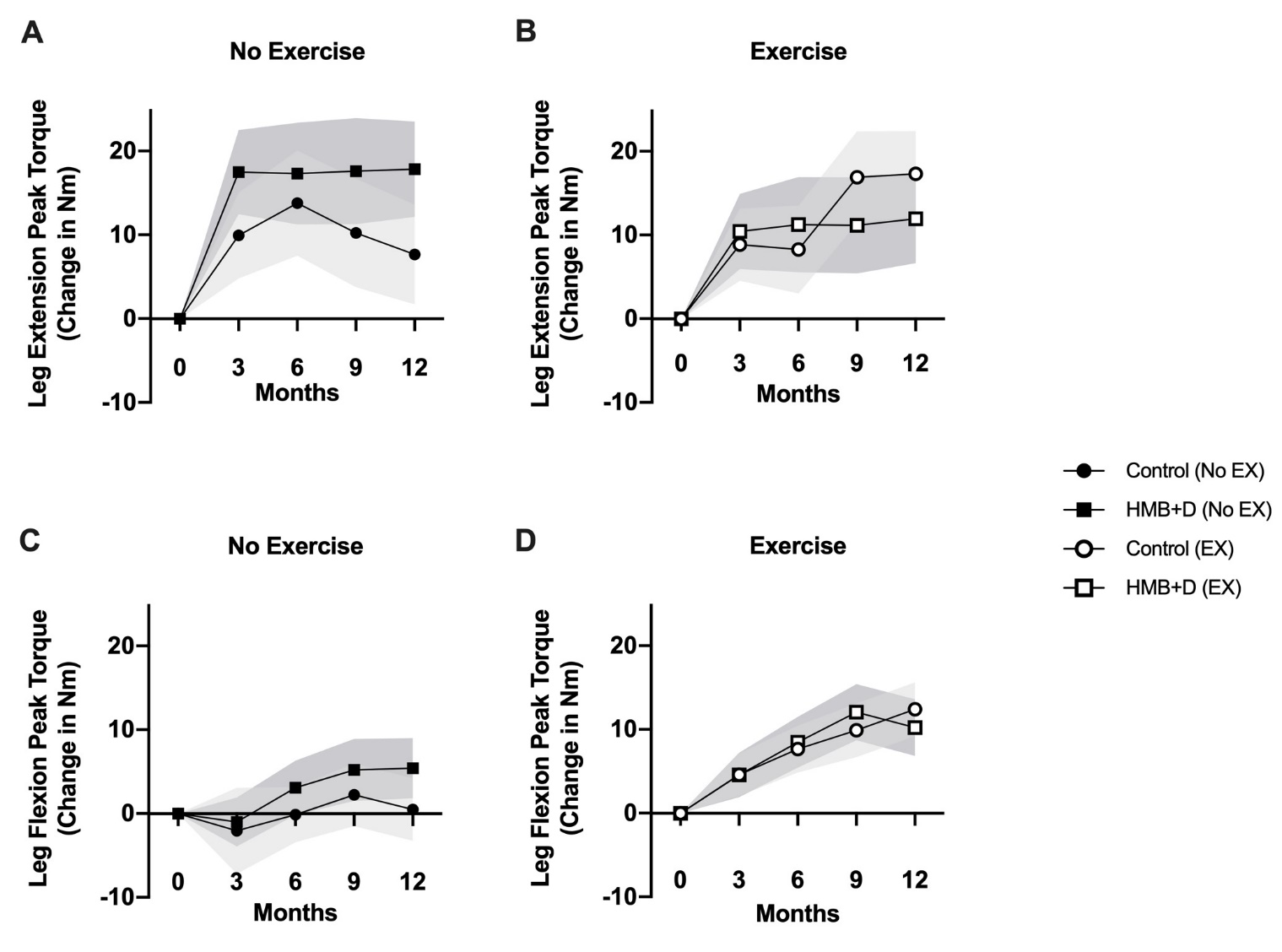


**Supplemental Figure 5:** Changes in total (sum right and left legs) peak torque at 90°/sec. Panels A (no exercise) and B (with exercise) represent knee extension, and panels C (with no exercise) and D (with exercise) represent knee flexion. There were main effects of exercise (*p*<0.05) on leg flexion peak torque at 3, 6, 9, and 12 months. Data are expressed as Mean ± SE (shaded area). HMB, calcium β-hydroxy-β-methylbutyrate. D, Vitamin D_3_. EX, Exercise.


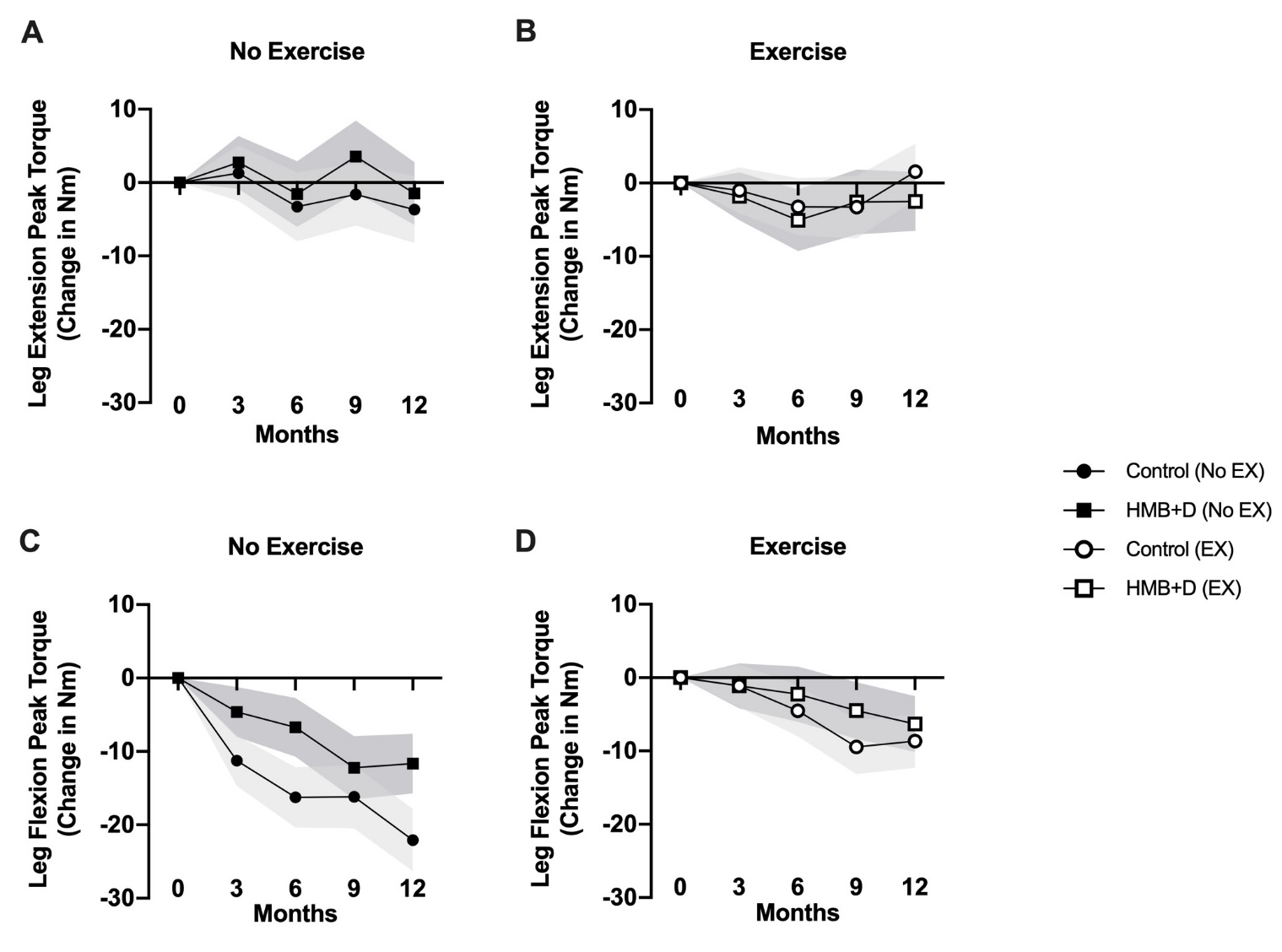


**Supplemental Figure 6:** Changes in total (sum right and left legs) peak torque at 180°/sec. Panels A (no exercise) and B (with exercise) represent knee extension, and panels C (with no exercise) and D (with exercise) represent knee flexion at 90°/sec. There were main effects of exercise (*p*<0.05) on leg flexion peak torque at 3, 6, and 12 months. Data are expressed as Mean ± SE (shaded area). HMB, calcium β-hydroxy-β-methylbutyrate. D, Vitamin D_3_. EX, Exercise.


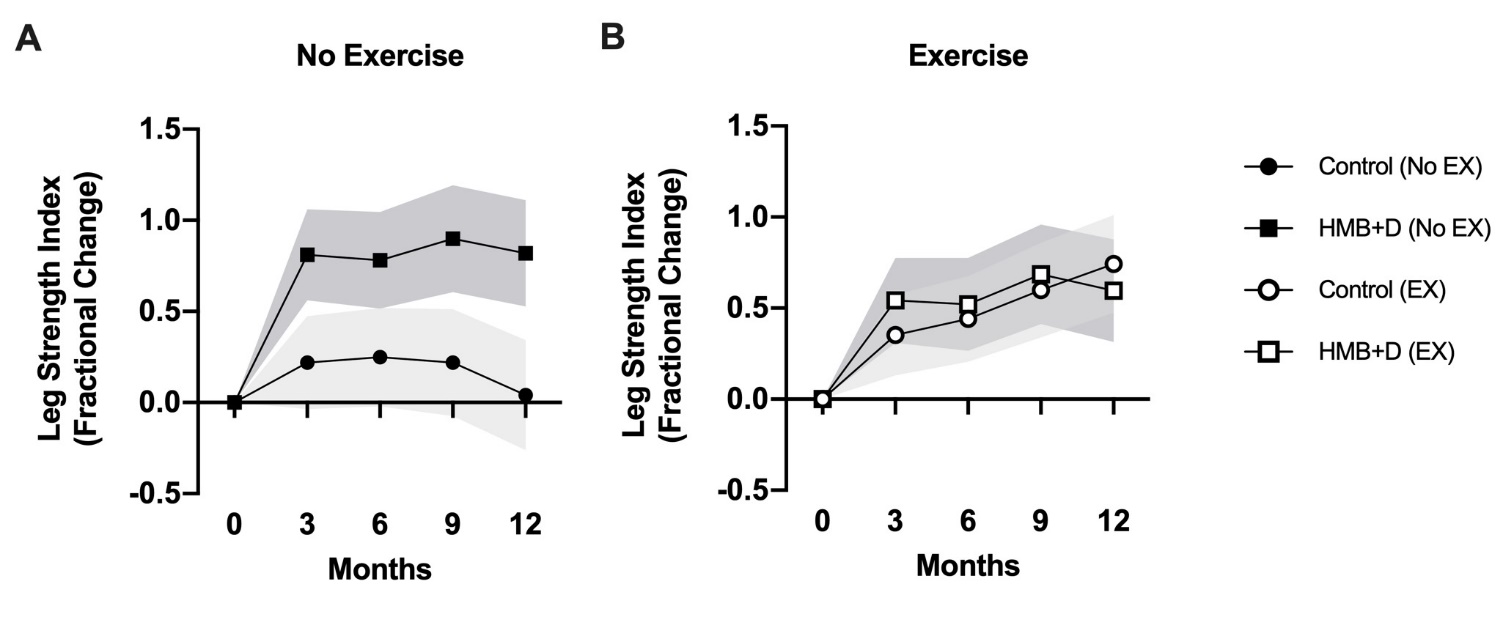


**Supplemental Figure 7.** Changes in Lower Composite Extremity Strength Index [(left leg extension peak torque at 60°/sec + 90°/sec + 180°/sec) + (right leg extension peak torque at 60°/sec + 90°/sec + 180°/sec) + (left leg flexion peak torque at 60°/sec + 90°/sec + 180°/sec) + (right leg flexion peak torque at 60°/sec + 90°/sec + 180°/sec)]. Data are expressed as Mean ± SE (shaded area). HMB, calcium β-hydroxy-β-methylbutyrate. D, Vitamin D_3_. EX, Exercise.


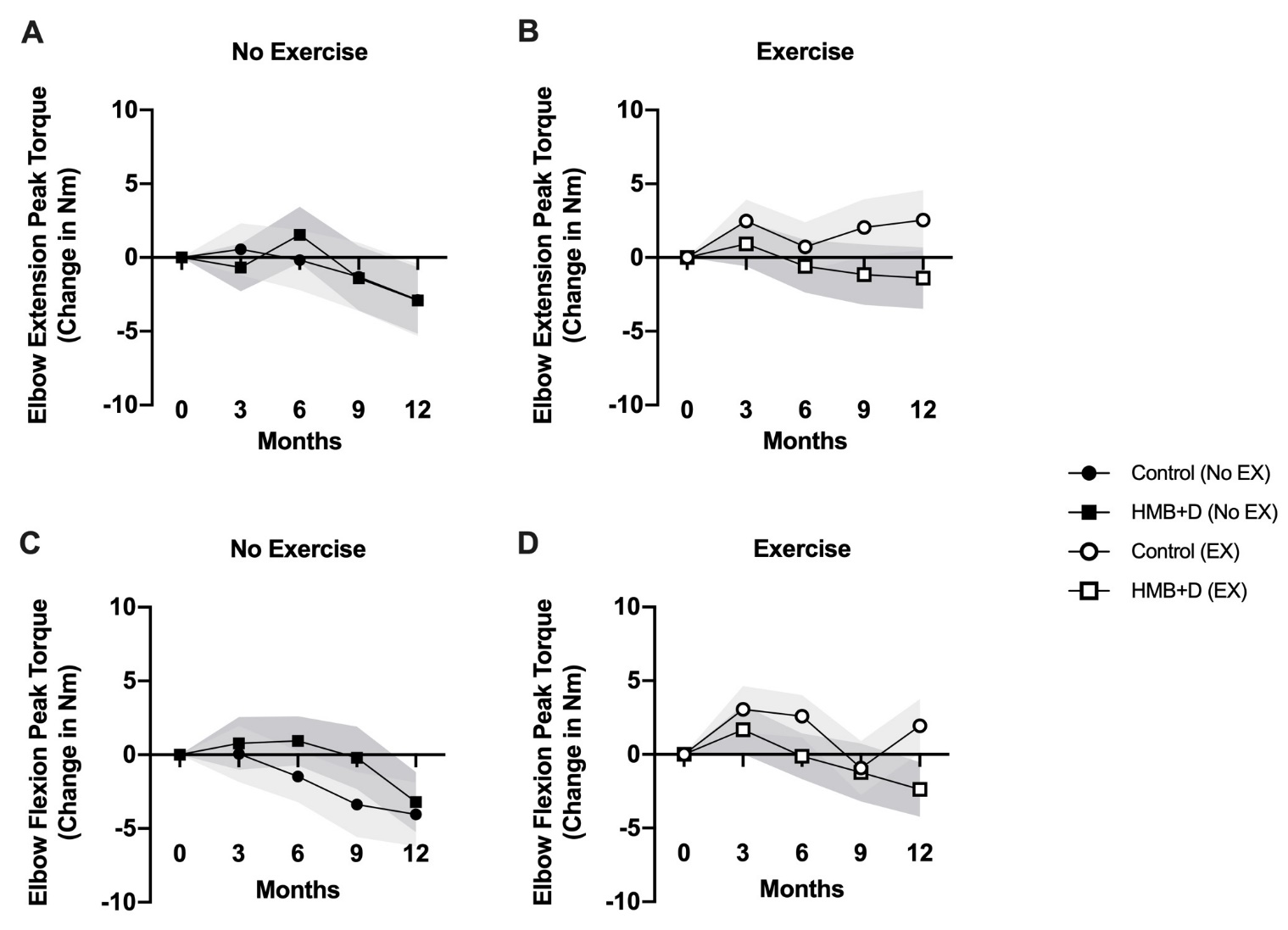


**Supplemental Figure 8:** Changes in total (sum right and left arms) peak torque at 60°/sec. Panels A (no exercise) and B (with exercise) represent elbow extension, and panels C (with no exercise) and D (with exercise) represent elbow flexion. Data are expressed as Mean ± SE (shaded area). HMB, calcium β-hydroxy-β-methylbutyrate. D, Vitamin D_3_. EX, Exercise.


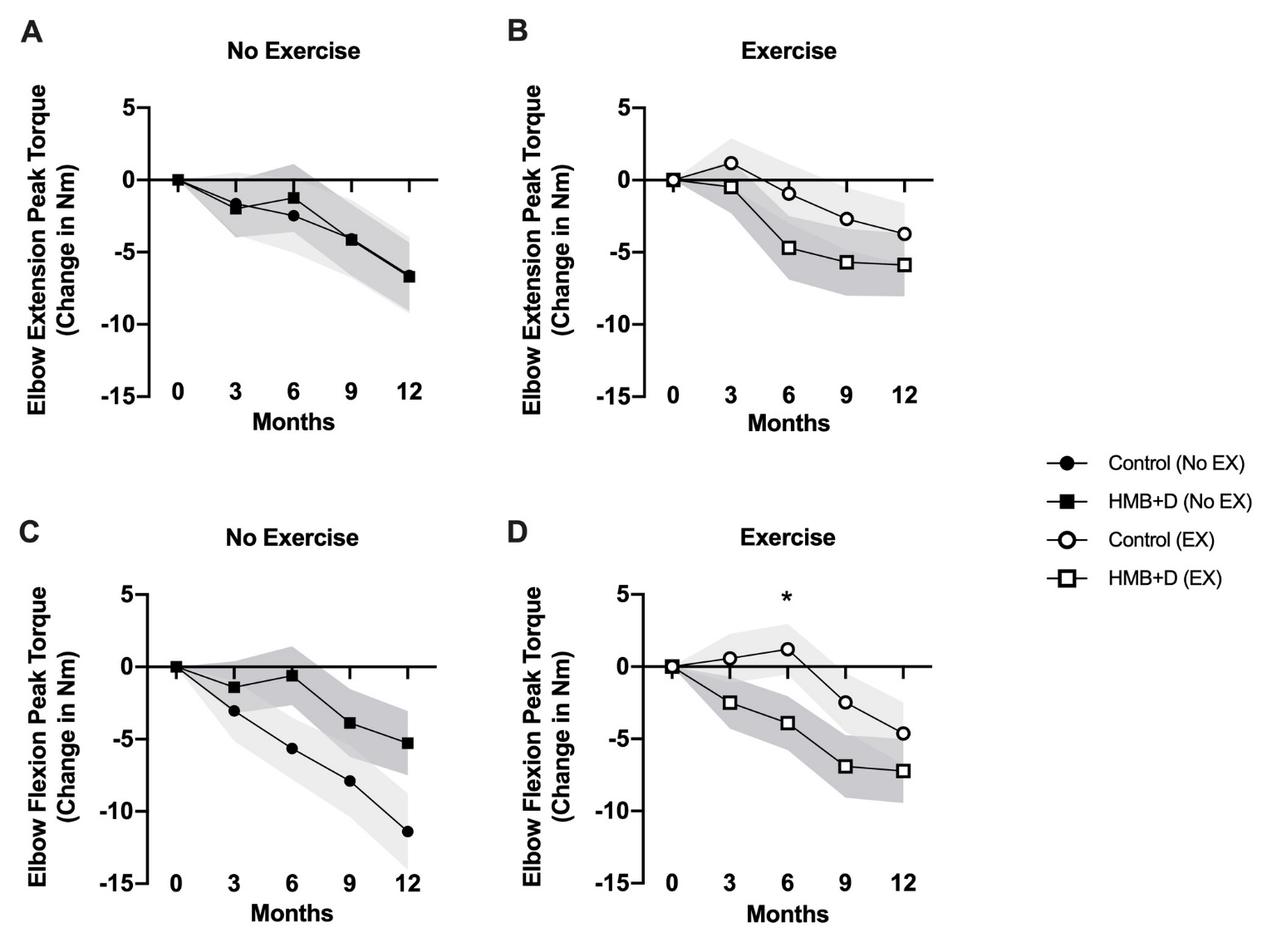
 **Supplemental Figure 9:** Changes in total (sum right and left arms) peak torque at 120°/sec. Panels A (no exercise) and B (with exercise) represent elbow extension, and panels C (with no exercise) and D (with exercise) represent elbow flexion. There was a significant treatment*exercise interaction for elbow flexion at 6 months. Post-hoc testing revealed significant differences between treatments within the exercise group (*) as well as a significant difference between exercise and no exercise within the control group. Data are expressed as Mean ± SE (shaded area). HMB, calcium β-hydroxy-β-methylbutyrate. D, Vitamin D_3_. EX, Exercise.


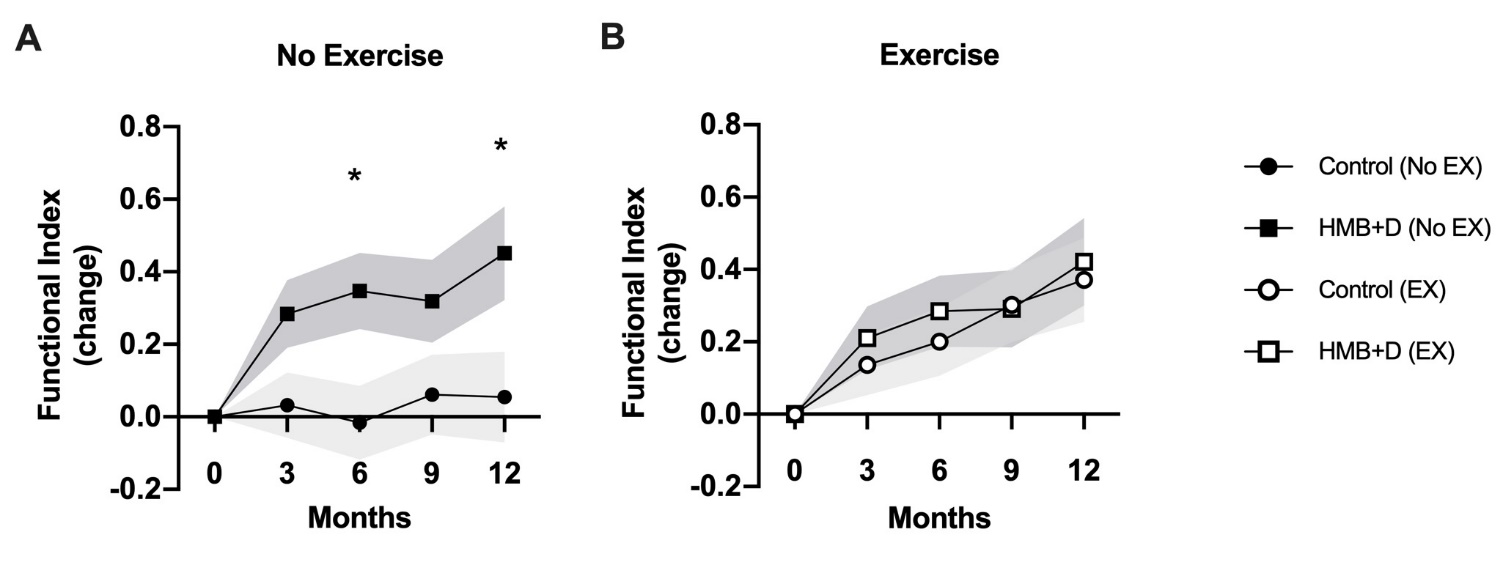


**Supplemental Figure 10.** Intent-to-treat analysis of effect of HMB+D supplementation on changes in composite functional index (sum of fractional improvement in Get Up, Get Up and Go, and right and left handgrip strength). There was a significant treatment main effect (*p*=0.02) of HMB+D supplementation at 6 months. *significant difference between HMB+D and control within group (no exercise or exercise); pre-planned contrast, *p*<0.05. Data are expressed as Mean ± SE (shaded area). HMB, calcium β-hydroxy-β-methylbutyrate. D, Vitamin D_3_. EX, Exercise.


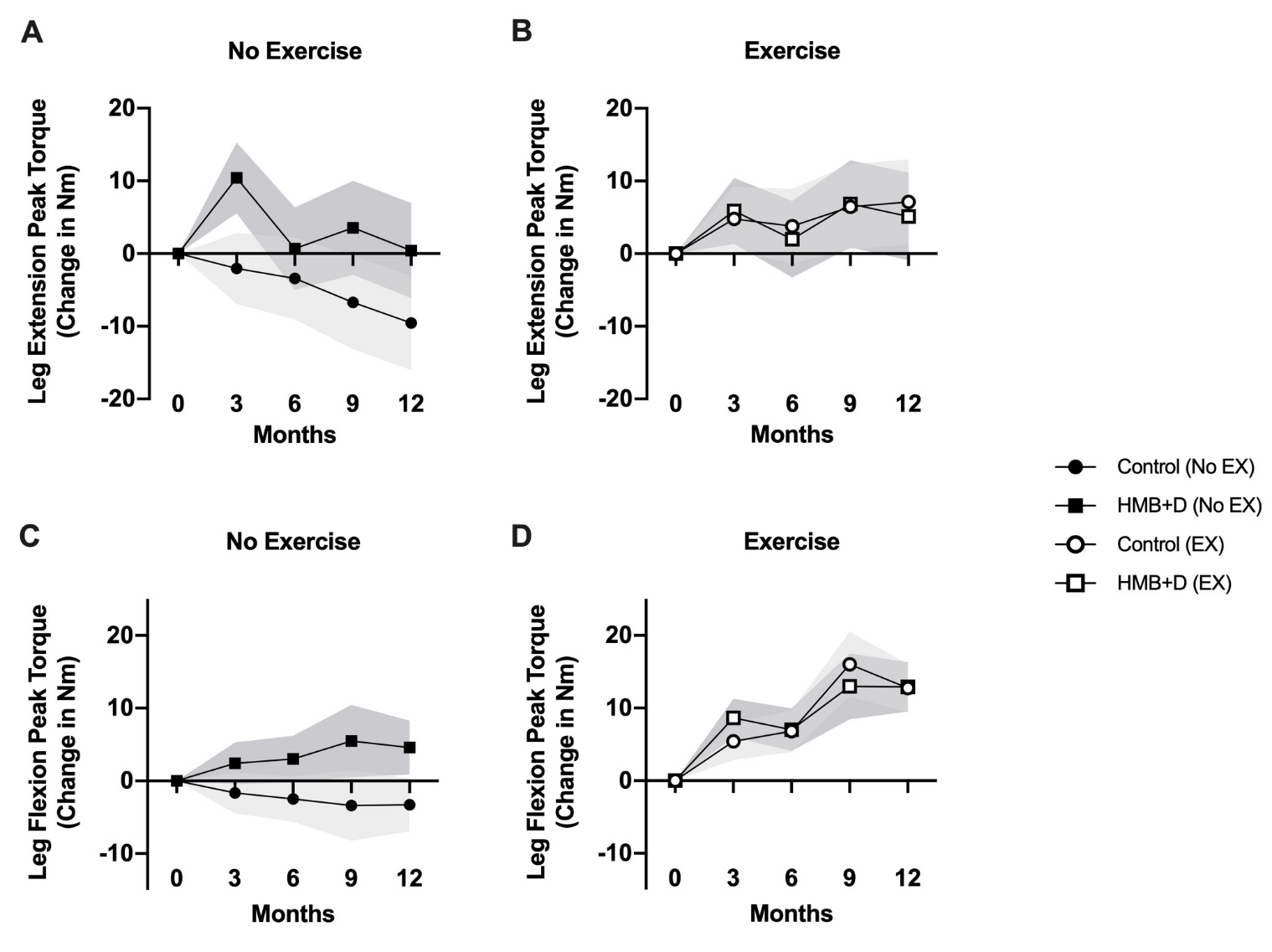


**Supplemental Figure 11:** Intent-to-treat analysis of effect of HMB+D supplementation on changes in total (sum right and left legs) peak torque at 60°/sec. Panels A (no exercise) and B (with exercise) represent knee extension, and panels C (no exercise) and D (with exercise) represent knee flexion. There were main effects of exercise (*p*<0.05) on leg flexion peak torque at 3, 6, 9, and 12 months. Data are expressed as Mean ± SE (shaded area). HMB, calcium β-hydroxy-β-methylbutyrate. D, Vitamin D_3_. EX, Exercise.


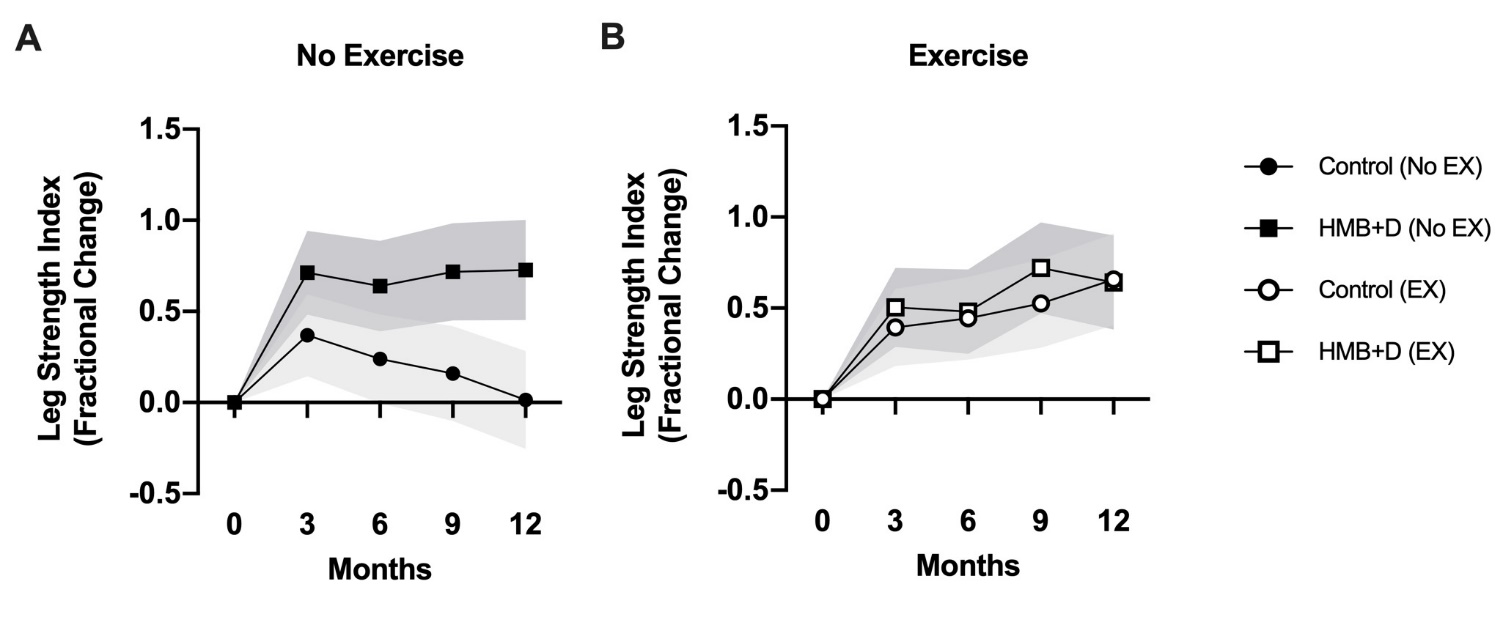


**Supplemental Figure 12**. Intent-to-treat analysis of effect of HMB+D supplementation on changes in lower composite extremity strength index [(left leg extension peak torque at 60°/sec + 90°/sec + 180°/sec) + (right leg extension peak torque at 60°/sec + 90°/sec + 180°/sec) + (left leg flexion peak torque at 60°/sec + 90°/sec + 180°/sec) + (right leg flexion peak torque at 60°/sec + 90°/sec + 180°/sec)]. Data are expressed as Mean ± SE (shaded area). HMB, calcium β-hydroxy-β-methylbutyrate. D, Vitamin D_3_. EX, Exercise.

*Supplemental Table 1. Compliance Data*

|  | HMB+D  (No EX) | Control  (No EX) | HMB+D  (EX) | Control  (EX) |
| --- | --- | --- | --- | --- |
| n | 27 | 26 | 30 | 34 |
| Capsules Consumed (%) | 97.2 ± 0.4 | 94.3 ± 1.4 | 96.4 ± 0.7 | 96.0 ± 0.7 |
| Exercise Compliance (%) | NA | NA | 83.2 ± 2.2 | 83.4 ± 2.0 |

Data are expressed as Mean ± SE. NA, not applicable. HMB, calcium β-hydroxy-β-methylbutyrate. D, Vitamin D_3_. EX, Exercise.

|  |  | |  |  |  |  |  |  |  |  |  |  |  |  |
| --- | --- | --- | --- | --- | --- | --- | --- | --- | --- | --- | --- | --- | --- | --- |
| **Supplemental Table 2.** Body composition changes after 6 and 12 months of supplementation with HMB+D or control with or without exercise. | | | | | | | | | | | | | |  |
|  | No Exercise | | | | | | | Exercise | | | | | |  |
|  | HMB+D | | | | Control | | | HMB+D | | | Control | | |  |
|  |  | Month | |  |  | Month |  |  | Month |  |  | Month |  | |
|  | 0 | 6 (Δ  from 0) | | 12 (Δ from 0) | 0 | 6 (Δ  from 0) | 12 (Δ from 0) | 0 | 6 (Δ  from 0) | 12 (Δ from 0) | 0 | 6 (Δ  from 0) | 12 (Δ from 0) | |
| Parameter | n=27 | n=27 | | n=27 | n=26 | n=26 | n=26 | n=30 | n=30 | n=30 | n=34 | n=34 | n=34 | |
| Weight, kg | 87.1 ± 3.9 | 0.56 ± 0.54 | | 0.02 ± 0.64 | 93.0 ± 2.9 | 0.27 ± 0.55 | 0.47 ± 0.66 | 86.9 ± 3.7 | 0.10 ± 0.51 | -0.21 ± 0.61 | 85.2 ± 3.0 | -0.35 ± 0.49 | -0.04 ± 0.58 | |
|  |  |  | |  |  |  |  |  |  |  |  |  |  | |
| DXA |  |  | |  |  |  |  |  |  |  |  |  |  | |
| Lean mass,  kg | 50.0 ± 2.2 | 0.44 ± 0.27 | | -0.14 ± 0.30 | 53.7 ± 1.7 | -0.33 ± 0.28 | -0.17 ± 0.31 | 50.4 ± 2.2 | 0.61 ± 0.26 | 0.36 ± 0.28 | 51.6 ± 2.0 | 0.49 ± 0.25 | 0.36 ± 0.27 | |
| Muscle mass,  kg | 47.5 ± 2.1 | 0.42 ± 0.28 | | -0.15 ± 0.30 | 51.0 ± 1.7 | -0.35 ± 0.28 | -0.21 ± 0.31 | 48.0 ± 2.1 | 0.58 ± 0.27 | 0.40 ± 0.29 | 49.2 ± 1.9 | 0.47 ± 0.25 | 0.35 ± 0.27 | |
| Fat mass,  kg | 34.6 ± 1.8 | 0.10 ± 0.41^c^ | | 0.04 ± 0.54 | 36.2 ± 1.9 | 0.82 ± 0.43^c^ | 0.76 ± 0.57 | 33.2 ± 1.9 | -0.59 ± 0.39^c^ | -0.63 ± 0.51 | 31.6 ± 1.5 | -0.71 ± 0.38^c^ | -0.57 ± 0.49 | |
| Body fat,  % | 40.7 ± 0.9 | 0.00 ± 0.30^c^ | | 0.21 ± 0.38^c^ | 40.1 ± 1.6 | 0.58 ± 0.31^c^ | 0.45 ± 0.40^c^ | 39.4 ± 1.2 | -0.63 ± 0.28^c^ | -0.59 ± 0.36^c^ | 37.9 ± 1.1 | -0.73 ± 0.27^c^ | -0.78 ± 0.34^c^ | |
| Trunk lean  mass, kg | 24.1 ± 1.1 | 0.25 ± 0.16^b^ | | 0.02 ± 0.19 | 25.9 ± 0.8 | -0.16 ± 0.16^b^ | 0.11 ± 0.19 | 24.4 ± 1.1 | 0.25 ± 0.15^b^ | 0.17 ± 0.17 | 24.6 ± 0.9 | -0.01 ± 0.14^b^ | 0.18 ± 0.17 | |
| Trunk fat  mass, kg | 17.7 ± 1.1 | 0.18 ± 0.26^c^ | | 0.09 ± 0.33 | 18.6 ± 0.9 | 0.53 ± 0.27^c^ | 0.38 ± 0.34 | 17.1 ± 1.1 | -0.39 ± 0.25^c^ | -0.39 ± 0.31 | 16.3 ± 0.9 | -0.56 ± 0.24^c^ | -0.28 ± 0.30 | |
| Trunk fat,  % | 41.5 ± 1.0 | 0.07 ± 0.39^c^ | | 0.13 ± 0.48^c^ | 41.5 ± 1.3 | 0.81 ± 0.41^c^ | 0.30 ± 0.49^c^ | 40.5 ± 1.2 | -0.72 ± 0.37^c^ | -0.58 ± 0.45^c^ | 39.3 ± 0.9 | -0.91 ± 0.35^c^ | -0.89 ± 0.43^c^ | |
| Appendicular  fat mass, kg | 15.6 ± 0.8 | -0.07 ± 0.19 | | -0.02 ± 0.25 | 16.3 ± 1.2 | 0.30 ± 0.20 | 0.44 ± 0.26 | 14.7 ± 0.9 | -0.18 ± 0.18 | -0.20 ± 0.24 | 13.9 ± 0.7 | -0.14 ± 0.17 | -0.26 ± 0.23 | |
| Appendicular  lean mass, kg | 22.0 ± 1.0 | 0.29 ± 0.17^c^ | | -0.01 ± 0.17^c^ | 23.9 ± 0.9 | -0.13 ± 0.17^c^ | -0.18± 0.17^c^ | 22.2 ± 1.1 | 0.37 ± 0.16^c^ | 0.26 ± 0.16^c^ | 23.2 ± 1.0 | 0.53 ± 0.15^c^ | 0.25 ± 0.15^c^ | |
| Bone mineral  density, g/cm^3^ | 1.15 ± 0.03 | -0.02 ± 0.02 | | -0.02 ± 0.02 | 1.20 ± 0.03 | -0.00 ± 0.02 | -0.01 ± 0.02 | 1.13 ± 0.03 | -0.04 ± 0.02 | -0.04 ± 0.02 | 1.24 ± 0.09 | -0.05 ± 0.02 | -0.04 ± 0.02 | |
|  |  |  | |  |  |  |  |  |  |  |  |  |  | |
| BIA |  |  | |  |  |  |  |  |  |  |  |  |  | |
| Lean mass, kg | 55.8 ± 2.4 | 1.31 ± 0.99 | | -0.20 ± 0.45^c^ | 59.8 ± 2.0 | -1.03 ± 1.03 | -0.63 ± 0.47^c^ | 56.2 ± 2.5 | 0.26 ± 0.95 | 0.79 ± 0.43^c^ | 56.2 ± 2.2 | 0.72 ± 0.91 | 0.39 ± 0.41^c^ | |
| Fat mass, kg | 31.4 ± 2.0 | 0.63 ± 0.84^c^ | | 0.08 ± 0.50^c^ | 33.1 ± 2.2 | 1.35 ± 0.90^c^ | 0.86 ± 0.54^c^ | 30.8 ± 1.7 | -0.75 ± 0.81^c^ | -0.94 ± 0.48^c^ | 29.3 ± 1.5 | -1.30 ± 0.76^c^ | -0.79 ± 0.45^c^ | |
| Body fat, % | 35.6 ± 1.2 | -0.32 ± 0.43^ac^ | | 0.26 ± 0.41^c^ | 35.5 ± 1.8 | 1.39 ± 0.46^ac^ | 0.83 ± 0.44^c^ | 35.2 ± 1.2 | -0.50 ± 0.42^ac^ | -0.92 ± 0.40^c^ | 34.2 ± 0.1 | -1.15 ± 0.39^ac^ | -0.85 ± 0.37^c^ | |
|  |  |  | |  |  |  |  |  |  |  |  |  |  | |
| BodPod |  |  | |  |  |  |  |  |  |  |  |  |  | |
| Lean mass, kg | 52.5 ± 2.3 | 0.81 ± 0.34 | | 0.48 ± 0.37 | 56.5 ± 2.1 | -0.01 ± 0.35 | -0.05 ± 0.38 | 53.3 ± 2.4 | 0.67 ± 0.32 | 0.71 ± 0.35 | 54.3 ± 2.0 | 0.95 ± 0.31 | 0.85 ± 0.33 | |
| Fat mass, kg | 33.7 ± 2.0 | -0.17 ± 0.58 | | -0.51 ± 0.70 | 35.1 ± 2.4 | 0.35 ± 0.59 | 0.52 ± 0.73 | 31.8 ± 2.1 | -0.60 ± 0.54 | -0.91 ± 0.67 | 30.4 ± 1.7 | -1.36 ± 0.52 | -0.99 ± 0.64 | |
| Body fat, % | 38.8 ± 1.2 | -0.37 ± 0.55 | | -0.64 ± 0.61 | 37.9 ± 2.1 | 0.20 ± 0.55 | 0.19 ± 0.63 | 36.9 ± 1.5 | -0.61 ± 0.50 | -0.90 ± 0.57 | 35.7 ± 1.3 | -1.39 ± 0.48 | -1.21 ± 0.54 | |

Participants consumed either the treatment [calcium β-hydroxy-β-methylbutyrate (HMB, 1.5 g) + Vitamin D_3_ (D, 1000 IU)] or a placebo control (calcium lactate) twice daily for 12 months. Data were analyzed by using a SAS Proc Mixed model of covariance analysis on the change at 3, 6, 9, and 12 months (6 and 12 months only for DXA). The model included the starting value as a covariate, sex, treatment, exercise, and treatment by exercise interaction. Only those subjects completing 12 months of study were included in the model. Data are presented as Mean ± SE.

DXA, dual energy x-ray absorptiometry; BIA, bioelectrical impedance analysis

a - treatment * exercise interaction (*p* < 0.05), b – main effect of treatment (*p* < 0.05), c – main effect of exercise (*p* < 0.05).

| **Supplemental Table 3.** Effect of HMB+D with and without exercise on parathyroid hormone and bone alkaline phosphatase levels in older adults. | | | | | | | | | | | | | | | | | | | | | |
| --- | --- | --- | --- | --- | --- | --- | --- | --- | --- | --- | --- | --- | --- | --- | --- | --- | --- | --- | --- | --- | --- |
|  | No Exercise | | | | | | | | | | Exercise | | | | | | | | | |  |
|  | HMB+D | | | | | Control | | | | | HMB+D | | | | | Control | | | | | Statistics |
|  |  | Month | |  | |  | Month | |  | |  | Month | |  | |  | Month | |  | |  |
|  | 0 | | 6 | | 12 | 0 | | 6 | | 12 | 0 | | 6 | | 12 | 0 | | 6 | | 12 |  |
| Parameter | (n=27) | | (n=27) | | (n=27) | (n=26) | | (n=26) | | (n=26) | (n=32) | | (n=30) | | (n=30) | (n=34) | | (n=34) | | (n=34) |  |
| Parathyroid hormone, pg/mL | 46.2 ± 21.3 | | 38.9 ± 15.0 | | 41.2 ± 22.7 | 46.3 ± 23.9 | | 39.3 ± 19.6 | | 45.4 ± 18.5 | 50.1 ± 24.5 | | 38.9 ± 22.6 | | 43.1 ± 25.1 | 43.8 ± 18.5 | | 44.2 ± 20.2 | | 42.1 ± 20.3 | d |
| Bone alkaline phosphatase, μg/L | 13.2 ± 5.5 | | 10.4 ± 2.9 | | 10.6 ± 3.2 | 12.8 ± 3.5 | | 10.8 ± 3.0 | | 11.1 ± 2.9 | 13.2 ± 4.4 | | 11.0 ± 3.7 | | 10.9 ± 3.2 | 11.7 ± 4.2 | | 10.3 ± 3.4 | | 10.4 ± 3.2 | a,d,e |
| Participants consumed either the treatment [calcium β-hydroxy-β-methylbutyrate (HMB,1.5 g) + Vitamin D_3_ (D,1000 IU)] or a placebo control (calcium lactate) twice daily for 12 months. Blood samples were taken at 0, 3, 6, 9, and 12 months of treatment and analyzed by a commercial laboratory (Heartland Assays, Ames, IA); data from 0, 6, and 12 months are presented. Data are presented as Mean ± SD. | | | | | | | | | | | | | | | | | | | | | |
| Significant effects based on probabilities from SAS Proc Mixed repeated measure model of analysis. The model included starting value, gender, treatment, exercise, time, treatment by exercise interaction, treatment by time interaction, exercise by time interaction, and treatment by exercise by time interaction. Only those participants completing 12 months of study were included in the model.  ^a^treatment main effect, ^b^exercise main effect, ^c^time main effect, ^d^treatment by exercise interaction, ^e^treatment by time interaction, ^f^exercise by time interaction, ^g^treatment by exercise by time interaction (*p* < 0.05 for all). | | | | | | | | | | | | | | | | | | | | | |

| **Supplemental Table 4.** Effect of HMB+D with and without exercise on blood chemistry values in older adults. | | | | | | | | | | | | | | |
| --- | --- | --- | --- | --- | --- | --- | --- | --- | --- | --- | --- | --- | --- | --- |
|  | No-Exercise | | | | | | | Exercise | | | | | |  |
|  | HMB+D | | | Control | | | HMB+D | | | | Control | | | Statistics |
|  | Month | | | Month | | | Month | | | | Month | | |  |
|  | 0 | 6 | 12 | 0 | 6 | 12 | 0 | | 6 | 12 | 0 | 6 | 12 |  |
| Parameter | (n=27) | (n=27) | (n=27) | (n=26) | (n=26) | (n=26) | (n=32) | | (n=30) | (n=30) | (n=34) | (n=34) | (n=34) |  |
| Creatinine Kinase, IU/L | 127 ± 67 | 119 ± 60 | 140 ± 84 | 126 ± 81 | 129 ± 92 | 156 ± 135 | 125 ± 68 | | 106 ± 50 | 115 ± 49 | 118 ± 56 | 142 ± 75 | 118 ± 64 | a |
| Glucose,  mg/dL | 101 ± 17 | 102 ± 16 | 106 ± 23 | 103 ± 25 | 102 ± 21 | 107 ± 33 | 101 ± 19 | | 102 ± 22 | 101 ± 15 | 100 ± 15 | 98 ±  14 | 99 ±  11 | b,d |
| Sodium,  nmol/L | 141 ±  2 | 142 ±  2 | 142 ±  2 | 141 ±  2 | 142 ±  2 | 142 ±  2 | 142 ±  2 | | 143 ±  3 | 143 ±  2 | 141 ±  2 | 142 ±  2 | 143 ±  3 | a,d |
| Potassium, nmol/L | 4.5 ± 0.3 | 4.9 ± 0.4 | 4.9 ± 0.4 | 4.7 ± 0.4 | 4.8 ± 0.4 | 4.8 ± 0.5 | 4.6 ± 0.3 | | 5.0 ± 0.5 | 4.8 ± 0.4 | 4.5 ± 0.4 | 4.8 ± 0.6 | 4.9 ± 0.4 | d |
| Chloride, nmol/L | 101 ±  2 | 102 ±  2 | 101 ±  2 | 101 ±  2 | 101 ±  2 | 102 ±  2 | 102 ±  2 | | 103 ±  3 | 103 ±  2 | 101 ±  2 | 102 ±  3 | 102 ±  3 | b,d |
| CO_2_,  mmol/L | 26.0 ± 2.8 | 26.6 ± 2.9 | 26.5 ± 2.0 | 26.6 ± 1.7 | 26.4 ± 1.7 | 25.8 ± 2.3 | 25.5 ± 2.0 | | 26.6 ± 2.4 | 26.7 ± 1.7 | 26.2 ± 1.9 | 26.2 ± 2.1 | 26.5 ± 2.1 |  |
| BUN,  mg/dL | 15.7 ± 4.1 | 17.2 ± 4.2 | 17.2 ± 4.2 | 16.5 ± 4.6 | 16.9 ± 4.3 | 16.0 ± 3.3 | 17.0 ± 4.0 | | 18.0 ± 3.0 | 17.4 ± 4.0 | 16.2 ± 5.0 | 17.1 ± 4.8 | 17.4 ± 5.6 | c,d,g |
| Creatinine, mg/dL | 0.90 ± 0.19 | 0.93 ± 0.18 | 0.98 ± 0.23 | 0.89 ± 0.17 | 0.91 ± 0.20 | 0.91 ± 0.18 | 0.91 ± 0.13 | | 0.93 ± 0.15 | 0.92 ± 0.15 | 0.93 ± 0.26 | 0.94 ± 0.26 | 0.93 ± 0.27 | d |
| BUN:  Creatinine | 17.9 ± 4.5 | 18.9 ± 5.1 | 18.1 ± 4.0 | 19.2 ± 5.7 | 19.0 ± 4.2 | 17.8 ± 3.7 | 18.9 ± 4.5 | | 19.9 ± 4.4 | 19.4 ± 5.0 | 17.9 ± 5.1 | 18.5 ± 4.9 | 19.3 ± 5.5 |  |
| Uric acid, mg/dL | 5.6 ± 1.4 | 6.0 ± 1.3 | 6.1 ± 1.5 | 5.6 ± 1.1 | 5.8 ± 1.0 | 5.5 ± 0.9 | 5.8 ± 1.3 | | 6.2 ± 1.3 | 6.0 ± 1.5 | 5.6 ± 0.9 | 5.4 ± 1.2 | 5.4 ± 1.2 | a,d,e |
| Calcium,  mg/dL | 9.4 ± 0.3 | 9.5 ± 0.4 | 9.7 ± 0.4 | 9.4 ± 0.3 | 9.5 ± 0.5 | 9.5 ± 0.4 | 9.3 ± 0.3 | | 9.5 ± 0.4 | 9.5 ± 0.4 | 9.4 ± 0.3 | 9.5 ± 0.4 | 9.5 ± 0.4 | a,d,g |
| Phosphorous, mg/dL | 3.4 ± 0.4 | 3.5 ± 0.5 | 3.6 ± 0.4 | 3.4 ± 0.5 | 3.5 ± 0.6 | 3.4 ± 0.5 | 3.5 ± 0.5 | | 3.5 ± 0.6 | 3.3 ± 0.5 | 3.4 ± 0.4 | 3.4 ± 0.6 | 3.4 ± 0.5 |  |
| Total Protein, g/dL | 6.8 ± 0.4 | 6.8 ± 0.4 | 6.8 ± 0.6 | 6.8 ± 0.4 | 6.8 ± 0.4 | 6.8 ± 0.4 | 6.7 ± 0.3 | | 6.8 ± 0.3 | 6.7 ± 0.3 | 6.7 ± 0.3 | 6.8 ± 0.3 | 6.8 ± 0.3 |  |
| Albumin,  g/dL | 4.4 ± 0.3 | 4.4 ± 0.2 | 4.4 ± 0.3 | 4.4 ± 0.2 | 4.4 ± 0.3 | 4.3 ± 0.2 | 4.4 ± 0.2 | | 4.4 ± 0.3 | 4.4 ± 0.3 | 4.4 ± 0.2 | 4.4 ± 0.2 | 4.3 ± 0.2 |  |
| Globulin,  g/dL | 2.4 ± 0.3 | 2.5 ± 0.3 | 2.5 ± 0.4 | 2.5 ± 0.4 | 2.4 ± 0.4 | 2.4 ± 0.4 | 2.3 ± 0.3 | | 2.5 ± 0.2 | 2.4 ± 0.3 | 2.3 ± 0.2 | 2.4 ± 0.3 | 2.5 ± 0.3 | d |
| Albumin:  Globulin | 1.8 ± 0.3 | 1.8 ± 0.3 | 1.8 ± 0.4 | 1.8 ± 0.4 | 1.9 ± 0.4 | 1.8 ± 0.3 | 1.9 ± 0.3 | | 1.8 ± 0.2 | 1.8 ± 0.3 | 2.0 ± 0.3 | 1.9 ± 0.3 | 1.8 ± 0.2 | d |
| Participants consumed either the treatment [calcium β-hydroxy-β-methylbutyrate (HMB, 1.5 g) + Vitamin D_3_ (D,1000 IU)] or a placebo (calcium lactate) twice daily for 12 months. Blood samples were taken at 0, 3, 6, 9, and 12 months of treatment and analyzed by a commercial laboratory (LabCorp, USA); data from 0, 6, and 12 months are presented. Data are presented as Mean ± SD. | | | | | | | | | | | | | | |
| Significant effects based on probabilities from SAS Proc Mixed repeated measure model of analysis. The model included starting value, gender, treatment, exercise, time, treatment by exercise interaction, treatment by time interaction, exercise by time interaction, and treatment by exercise by time interaction. Only those participants completing 12 months of study were included in the model.  ^a^treatment main effect, ^b^exercise main effect, ^c^time main effect, ^d^treatment by exercise interaction, ^e^treatment by time interaction, ^f^exercise by time interaction, ^g^treatment by exercise by time interaction (*p* < 0.05 for all). | | | | | | | | | | | | | | |

| **Supplemental Table 5.** Effect of HMB+D with and without exercise on blood lipid values in older adults. | | | | | | | | | | | | | | |
| --- | --- | --- | --- | --- | --- | --- | --- | --- | --- | --- | --- | --- | --- | --- |
|  | No-Exercise | | | | | | | Exercise | | | | | |  |
|  | HMB+D | | | Control | | | HMB+D | | | | Control | | | Statistics |
|  | Month | | | Month | | | Month | | | | Month | | |  |
|  | 0 | 6 | 12 | 0 | 6 | 12 | 0 | | 6 | 12 | 0 | 6 | 12 |  |
| Parameter | (n=27) | (n=27) | (n=27) | (n=26) | (n=26) | (n=26) | (n=32) | | (n=30) | (n=30) | (n=34) | (n=34) | (n=34) |  |
| Triglycerides, mg/dL | 133 ± 68 | 129 ± 59 | 126 ± 59 | 133 ± 62 | 124 ± 58 | 156 ± 144 | 122 ± 51 | | 134 ± 62 | 139 ± 70 | 115 ± 38 | 117 ± 52 | 115 ± 43 |  |
| Total cholesterol, mg/dL | 198 ± 44 | 197 ± 45 | 195 ± 42 | 186 ± 33 | 180 ± 38 | 181 ± 47 | 206 ± 50 | | 201 ± 33 | 197 ± 30 | 202 ± 31 | 202 ± 32 | 199 ± 36 |  |
| HDL,  mg/dL | 58 ±  21 | 57 ±  21 | 59 ±  21 | 53 ±  15 | 55 ±  16 | 54 ±  17 | 56 ±  13 | | 56 ±  12 | 57 ±  13 | 58 ±  15 | 61 ±  16 | 61 ±  15 |  |
| VLDL,  mg/dL | 27 ±  14 | 26 ±  12 | 25 ±  12 | 26 ±  12 | 25 ±  12 | 27 ±  18 | 25 ±  10 | | 27 ±  12 | 28 ±  14 | 23 ±  8 | 23 ±  11 | 23 ±  9 |  |
| LDL,  mg/dL | 113 ± 39 | 114 ± 37 | 111 ± 35 | 107 ± 32 | 101 ± 34 | 94 ±  31 | 126 ± 46 | | 118 ± 31 | 112 ± 27 | 121 ± 27 | 117 ± 27 | 115 ± 33 | d |
| LDL/HDL  Ratio | 2.1 ± 0.8 | 2.2 ± 0.8 | 2.1 ± 0.9 | 2.1 ± 0.7 | 2.0 ± 0.9 | 1.8 ± 0.6 | 2.4 ± 1.0 | | 2.2 ± 0.7 | 2.1 ± 0.6 | 2.2 ± 0.8 | 2.1 ± 0.8 | 2.0 ± 0.9 | d |
| Participants consumed either the treatment [calcium β-hydroxy-β-methylbutyrate (HMB, 1.5 g) + Vitamin D_3_ (D,1000 IU)] or a placebo (calcium lactate) twice daily for 12 months. Blood samples were taken at 0, 3, 6, 9, and 12 months of treatment and analyzed by a commercial laboratory (LabCorp, USA); data from 0, 6, and 12 months are presented. Data are presented as Mean ± SD. | | | | | | | | | | | | | | |
| Significant effects based on probabilities from SAS Proc Mixed repeated measure model of analysis. The model included starting value, gender, treatment, exercise, time, treatment by exercise interaction, treatment by time interaction, exercise by time interaction, and treatment by exercise by time interaction. Only those participants completing 12 months of study were included in the model.  ^a^treatment main effect, ^b^exercise main effect, ^c^time main effect, ^d^treatment by exercise interaction, ^e^treatment by time interaction, ^f^exercise by time interaction, ^g^treatment by exercise by time interaction (*p* < 0.05 for all). | | | | | | | | | | | | | | |

| **Supplemental Table 6.** Effect of HMB+D with and without exercise on markers of hepatic function in older adults. | | | | | | | | | | | | | | |
| --- | --- | --- | --- | --- | --- | --- | --- | --- | --- | --- | --- | --- | --- | --- |
|  | No-Exercise | | | | | | Exercise | | | | | | |  |
|  | HMB+D | | | Control | | | | HMB+D | | | Control | | | Statistics |
|  | Month | | | Month | | | | Month | | | Month | | |  |
|  | 0 | 6 | 12 | 0 | 6 | 12 | | 0 | 6 | 12 | 0 | 6 | 12 |  |
| Parameter | (n=27) | (n=27) | (n=27) | (n=26) | (n=26) | (n=26) | | (n=32) | (n=30) | (n=30) | (n=34) | (n=34) | (n=34) |  |
| Total  Bilirubin,  mg/dL | 0.69 ± 0.38 | 0.60 ± 0.23 | 0.64 ± 0.28 | 0.56 ± 0.22 | 0.55 ± 0.21 | 0.53 ± 0.20 | | 0.54 ± 0.23 | 0.52 ± 0.28 | 0.52 ± 0.24 | 0.60 ± 0.32 | 0.56 ± 0.24 | 0.52 ± 0.24 |  |
| Alkaline phosphatase (ALP),  IU/L | 72 ±  20 | 70 ±  21 | 70 ±  22 | 73 ±  18 | 68 ±  16 | 71 ±  17 | | 73 ±  17 | 68 ±  15 | 68 ±  13 | 72 ±  22 | 66 ±  20 | 67 ±  21 | d,f |
| Lactate dehydrogenase, IU/L | 182 ± 40 | 185 ± 30 | 184 ± 33 | 178 ± 23 | 176 ± 23 | 179 ± 27 | | 176 ± 35 | 168 ± 28 | 172 ± 29 | 172 ± 21 | 178 ± 21 | 171 ± 18 | b |
| Aspartate aminotransferase (AST), IU/L | 21 ±  5 | 20 ±  5 | 22 ±  5 | 22 ±  6 | 19 ±  5 | 21 ±  5 | | 21 ±  6 | 21 ±  5 | 22 ±  5 | 22 ±  7 | 23 ±  7 | 23 ±  7 |  |
| Alanine aminotransferase (ALT), IU/L | 21 ±  9 | 21 ±  11 | 21 ±  11 | 20 ±  7 | 19 ±  7 | 19 ±  7 | | 19 ± 6 | 19 ±  7 | 19 ±  7 | 21 ±  9 | 20 ±  6 | 20 ±  7 |  |
| Gamma-glutamyltransferase, (GGT) IU/L | 25 ±  15 | 24 ±  14 | 25 ±  15 | 25 ±  16 | 25 ±  15 | 24 ±  16 | | 23 ±  15 | 23 ±  14 | 23 ±  15 | 25 ±  13 | 24 ±  11 | 24 ±  12 |  |
| Iron,  μg/dL | 107 ± 32 | 98 ±  25 | 106 ± 26 | 102 ± 39 | 98 ±  29 | 94 ±  35 | | 101 ± 34 | 96 ±  33 | 104 ± 36 | 98 ±  36 | 96 ±  24 | 96 ±  28 |  |
| Participants consumed either the treatment [calcium β-hydroxy-β-methylbutyrate (HMB, 1.5 g) + Vitamin D_3_ (D,1000 IU)] or a placebo (calcium lactate) twice daily for 12 months. Blood samples were taken at 0, 3, 6, 9, and 12 months of treatment and analyzed by a commercial laboratory (LabCorp, USA); data from 0, 6, and 12 months are presented. Data are presented as Mean ± SD. | | | | | | | | | | | | | | |
| Significant effects based on probabilities from SAS Proc Mixed repeated measure model of analysis. The model included starting value, gender, treatment, exercise, time, treatment by exercise interaction, treatment by time interaction, exercise by time interaction, and treatment by exercise by time interaction. Only those participants completing 12 months of study were included in the model.  ^a^treatment main effect, ^b^exercise main effect, ^c^time main effect, ^d^treatment by exercise interaction, ^e^treatment by time interaction, ^f^exercise by time interaction, ^g^treatment by exercise by time interaction (*p*<0.05 for all). | | | | | | | | | | | | | | |

| **Supplemental Table 7.** Effect of HMB+D with and without exercise on hematology values in older adults. | | | | | | | | | | | | | | |
| --- | --- | --- | --- | --- | --- | --- | --- | --- | --- | --- | --- | --- | --- | --- |
|  | No-Exercise | | | | | | Exercise | | | | | | |  |
|  | HMB+D | | | Control | | | | HMB+D | | | Control | | | Statistics |
|  | Month | | | Month | | | | Month | | | Month | | |  |
|  | 0 | 6 | 12 | 0 | 6 | 12 | | 0 | 6 | 12 | 0 | 6 | 12 |  |
| Parameter | (n=27) | (n=27) | (n=27) | (n=26) | (n=26) | (n=26) | | (n=32) | (n=30) | (n=30) | (n=34) | (n=34) | (n=34) |  |
| White blood cells, cells x 10^3^/µL | 6.2 ± 1.5 | 6.1 ± 1.3 | 6.0 ± 1.6 | 5.8 ± 1.3 | 5.8 ± 1.0 | 5.9 ± 1.5 | | 6.0 ± 1.4 | 5.8 ± 1.5 | 5.8 ± 1.5 | 5.7 ± 1.5 | 5.5 ± 1.1 | 5.4 ± 1.1 |  |
| Red blood cells, cells x 10^6^/µL | 4.7 ± 0.2 | 4.7 ± 0.2 | 4.7 ± 0.3 | 4.8 ± 0.4 | 4.8 ± 0.4 | 4.8 ± 0.5 | | 4.8 ± 0.4 | 4.8 ± 0.4 | 4.7 ± 0.4 | 4.8 ± 0.4 | 4.7 ± 0.4 | 4.7 ± 0.4 |  |
| Hemoglobin,  g/dL | 14.5 ± 1.0 | 14.4 ± 1.0 | 14.5 ± 1.1 | 14.4 ± 1.5 | 14.3 ± 4.5 | 14.4 ± 1.8 | | 14.1 ± 1.2 | 14.2 ± 1.2 | 14.1 ± 1.2 | 14.5 ± 1.1 | 14.2 ± 1.2 | 14.3 ± 1.3 |  |
| Hematocrit,  % | 43 ±  3 | 43 ±  3 | 43 ±  3 | 43 ±  4 | 43 ±  4 | 43 ±  5 | | 42 ±  3 | 43 ±  3 | 43 ±  3 | 43 ±  3 | 43 ±  3 | 43 ±  3 |  |
| Mean corpuscular volume, fL | 92 ±  4 | 92 ±  4 | 92 ±  4 | 90 ±  4 | 90 ±  6 | 90 ±  6 | | 90 ±  7 | 90 ±  7 | 91 ±  7 | 90 ±  4 | 92 ±  5 | 91 ±  4 | d |
| Mean corpuscular hemoglobin, pg | 31 ±  2 | 31 ±  1 | 31 ±  2 | 30 ±  2 | 30 ±  2 | 30 ±  2 | | 30 ±  3 | 30 ±  3 | 30 ±  3 | 30 ±  2 | 30 ±  2 | 31 ±  2 |  |
| Mean cell hemoglobin, g/dL | 34 ±  1 | 33 ±  1 | 33 ±  1 | 33 ±  1 | 33 ±  1 | 33 ±  1 | | 33 ±  1 | 33 ±  1 | 33 ±  1 | 34 ±  1 | 33 ±  1 | 33 ±  1 | d,e,g |
| Platelets,  x 10^3^/µL | 245 ± 57 | 246 ± 64 | 250 ± 53 | 242 ± 48 | 244 ± 51 | 248 ± 53 | | 249 ± 57 | 249 ± 62 | 247 ± 58 | 222 ± 42 | 238 ± 53 | 227 ± 49 |  |
| RDW,  % | 13.8 ± 0.6 | 13.8 ± 0.6 | 13.7 ± 0.7 | 13.8 ± 0.5 | 14.0 ± 1.0 | 14.0 ± 1.1 | | 13.9 ± 1.4 | 13.9 ± 1.3 | 13.9 ± 1.0 | 13.9 ± 0.9 | 13.9 ± 1.0 | 13.9 ± 0.9 |  |
| Neutrophils,  x 10^3^/µL | 3.5 ± 1.2 | 3.6 ± 1.1 | 3.3 ± 1.1 | 3.3 ± 1.0 | 3.3 ± 0.8 | 3.4 ± 1.1 | | 3.3 ± 1.0 | 3.2 ± 0.9 | 3.2 ± 0.8 | 3.2 ± 1.2 | 3.1 ± 0.9 | 2.8 ± 0.9 | b |
| Lymphocytes,  x 10^3^/µL | 1.8 ± 0.5 | 1.7 ± 0.6 | 1.9 ± 0.7 | 1.7 ± 0.6 | 1.7 ± 0.5 | 1.7 ± 0.5 | | 1.9 ± 0.7 | 2.0 ± 0.7 | 1.9 ± 0.7 | 1.8 ± 0.4 | 1.7 ± 0.4 | 1.9 ± 0.6 |  |
| Monocytes,  x 10^3^/µL | 0.56 ± 0.17 | 0.60 ± 0.20 | 0.58 ± 0.22 | 0.52 ± 0.15 | 0.48 ± 0.12 | 0.50 ± 0.15 | | 0.53 ± 0.17 | 0.52 ± 0.15 | 0.54 ± 0.17 | 0.58 ± 0.21 | 0.52 ± 0.13 | 0.55 ± 0.13 |  |
| Eosinophils,  x 10^3^/µL | 0.18 ± 0.10 | 0.20 ± 0.10 | 0.20 ± 0.13 | 0.19 ± 0.13 | 0.20 ± 0.14 | 0.22 ± 0.13 | | 0.25 ± 0.14 | 0.18 ± 0.09 | 0.21 ± 0.12 | 0.16 ± 0.11 | 0.18 ± 0.13 | 0.17 ± 0.10 |  |
| Basophils,  x 10^3^/µL | 0.02 ± 0.04 | 0.02 ± 0.04 | 0.04 ± 0.05 | 0.02 ± 0.04 | 0.03 ± 0.04 | 0.03 ± 0.04 | | 0.02 ± 0.04 | 0.02 ± 0.05 | 0.02 ± 0.04 | 0.02 ± 0.04 | 0.02 ± 0.04 | 0.03 ± 0.05 |  |
| Neutrophils,  % | 58 ±  7 | 58 ±  8 | 54 ±  7 | 57 ±  9 | 57 ±  9 | 57 ±  7 | | 55 ±  7 | 54 ±  6 | 54 ±  7 | 55 ±  7 | 55 ±  8 | 51 ±  11 | g |
| Lymphocytes,  % | 29 ±  6 | 29 ±  8 | 31 ±  7 | 30 ±  8 | 30 ±  8 | 30 ±  7 | | 31 ±  8 | 33 ±  6 | 33 ±  7 | 32 ±  7 | 32 ±  8 | 35 ±  10 | g |
| Monocytes,  % | 9.6 ± 2.9 | 9.6 ± 2.6 | 9.9 ± 3.3 | 9.1 ± 2.7 | 8.5 ± 2.1 | 8.6 ± 2.3 | | 8.8 ± 2.0 | 8.8 ± 1.9 | 9.2 ± 1.5 | 10.2 ± 2.3 | 9.7 ± 2.0 | 10.3 ± 3.0 |  |
| Eosinophils,  % | 3.0 ± 1.8 | 3.3 ± 1.9 | 3.7 ± 2.7 | 3.3 ± 2.1 | 3.5 ± 2.2 | 3.8 ± 2.4 | | 4.0 ± 1.9 | 3.2 ± 1.5 | 3.6 ± 1.7 | 2.9 ± 1.9 | 3.1 ± 2.0 | 3.0 ± 1.9 |  |
| Basophils,  % | 0.71 ± 1.01 | 0.65 ± 0.56 | 0.93 ± 0.55 | 0.68 ± 0.63 | 0.63 ± 0.58 | 0.58 ± 0.50 | | 0.72 ± 0.52 | 0.86 ± 0.79 | 0.69 ± 0.54 | 0.56 ± 0.50 | 0.61 ± 0.50 | 0.75 ± 0.72 | g |
| Participants consumed either the treatment [calcium β-hydroxy-β-methylbutyrate (HMB, 1.5 g) + Vitamin D_3_ (D,1000 IU)] or a placebo (calcium lactate) twice daily for 12 months. Blood samples were taken at 0, 3, 6, 9, and 12 months of treatment and analyzed by a commercial laboratory (LabCorp, USA); data from 0, 6, and 12 months are presented. Data are presented as Mean ± SD. | | | | | | | | | | | | | | |
| Significant effects based on probabilities from SAS Proc Mixed repeated measure model of analysis. The model included starting value, gender, treatment, exercise, time, treatment by exercise interaction, treatment by time interaction, exercise by time interaction, and treatment by exercise by time interaction. Only those participants completing 12 months of study were included in the model.  ^a^treatment main effect, ^b^exercise main effect, ^c^time main effect, ^d^treatment by exercise interaction, ^e^treatment by time interaction, ^f^exercise by time interaction, ^g^treatment by exercise by time interaction (*p* < 0.05 for all). | | | | | | | | | | | | | | |

| **Supplemental Table 8.** Effect of HMB+D with and without exercise on resting heart rate and blood pressure in older adults. | | | | | | | | | | | | | |
| --- | --- | --- | --- | --- | --- | --- | --- | --- | --- | --- | --- | --- | --- |
|  | No-Exercise | | | | | | Exercise | | | | | |  |
|  | HMB+D | | | Control | | | HMB+D | | | Control | | | Statistics |
|  | Month | | | Month | | | Month | | | Month | | |  |
|  | 0 | 6 | 12 | 0 | 6 | 12 | 0 | 6 | 12 | 0 | 6 | 12 |  |
| Parameter | (n=27) | (n=26) | (n=27) | (n=26) | (n=26) | (n=26) | (n=30) | (n=30) | (n=30) | (n=34) | (n=34) | (n=34) |  |
| Heart rate,  bpm | 64.3 ± 8.7 | 64.1 ± 8.4 | 63.4 ± 10.0 | 66.4 ± 10.6 | 67.0 ± 11.1 | 67.8 ± 11.6 | 66.6 ± 10.0 | 67.2 ± 11.9 | 67.0 ± 10.6 | 63.9 ± 8.9 | 62.5 ± 7.7 | 62.0 ± 8.0 |  |
| Systolic blood pressure, mmHg | 138 ± 19 | 142 ± 19 | 139 ± 21 | 143 ± 21 | 141 ± 18 | 139 ± 22 | 140 ± 16 | 133 ± 15 | 135 ± 16 | 139 ± 17 | 137 ± 18 | 132 ± 17 | b,d |
| Diastolic blood pressure, mmHg | 76 ±  10 | 76 ±  8 | 75 ±  7 | 79 ±  8 | 78 ±  8 | 76 ±  9 | 77 ±  9 | 74 ±  8 | 75 ±  10 | 79 ±  10 | 75 ±  10 | 73 ±  8 | b,d,f |
| Mean Arterial Pressure, mmHg | 97 ±  11 | 98 ±  10 | 96 ±  10 | 100 ± 11 | 99 ±  10 | 97 ±  11 | 98 ±  10 | 93 ±  9 | 95 ±  10 | 99 ±  10 | 95 ±  11 | 93 ±  9 | b,d,f |
| Participants consumed either the treatment [calcium β-hydroxy-β-methylbutyrate (HMB, 1.5 g) + Vitamin D_3_ (D,1000 IU)] or a placebo (calcium lactate) twice daily for 12 months. . Resting heart rate and blood pressure were measured at 0, 3, 6, 9, and 12 months of treatment; data from 0, 6, and 12 months are presented. Data are presented as Mean ± SD. | | | | | | | | | | | | | |
| Significant effects based on probabilities from SAS Proc Mixed repeated measure model of analysis. The model included starting value, gender, treatment, exercise, time, treatment by exercise interaction, treatment by time interaction, exercise by time interaction, and treatment by exercise by time interaction. Only those participants completing 12 months of study were included in the model.  ^a^treatment main effect, ^b^exercise main effect, ^c^time main effect, ^d^treatment by exercise interaction, ^e^treatment by time interaction, ^f^exercise by time interaction, ^g^treatment by exercise by time interaction (*p*< 0.05 for all). | | | | | | | | | | | | | |

| **Supplemental Table 9.** Effect of HMB+D with and without exercise on emotional profile of older adults based on Circumplex Affect model. | | | | | | | | | | | | | | |
| --- | --- | --- | --- | --- | --- | --- | --- | --- | --- | --- | --- | --- | --- | --- |
|  | No-Exercise | | | | | | Exercise | | | | | | |  |
|  | HMB+D | | | Control | | | HMB+D | | | Control | | | | Statistics |
|  | Month | | | Month | | | Month | | | Month | | | |  |
|  | 0 | 6 | 12 | 0 | 6 | 12 | 0 | 6 | 12 | | 0 | 6 | 12 |  |
| Parameter | (n=  26-27) | (n=  26) | (n=  26) | (n=  24-26) | (n=  25-26) | (n=  25-26) | (n=  28-30) | (n=  29-30) | (n=  29-30) | | (n=  31-32) | (n=  34) | (n=  34) |  |
| High  Activation | 12.6 ± 3.6 | 13.7 ± 3.2 | 14.2 ± 3.1 | 13.7 ± 4.3 | 12.8 ± 3.8 | 13.9 ± 3.1 | 15.1 ± 4.1 | 15.5 ± 4.8 | 15.6 ± 4.7 | | 14.2 ± 3.8 | 13.8 ± 4.1 | 14.0 ±3.9 | a,e |
| Activated Pleasant Affect | 16.3 ± 4.9 | 16.7 ± 3.5 | 17.0 ± 5.2 | 17.0 ± 4.5 | 15.7 ± 4.5 | 16.2 ± 4.3 | 17.7 ± 4.7 | 18.7 ± 4.9 | 18.9 ± 5.3 | | 17.1 ± 5.6 | 17.1 ± 5.4 | 17.7 ± 5.5 | a |
| Unactivated Pleasant Affect | 22.9 ± 3.5 | 21.6 ± 4.3 | 22.3 ± 4.9 | 21.6 ± 3.6 | 21.0 ± 3.2 | 20.5 ± 2.9 | 22.3 ± 4.2 | 22.9 ± 3.6 | 23.3 ± 3.6 | | 20.9 ± 4.2 | 21.8 ± 3.8 | 21.8 ± 3.9 | b,f |
| Pleasant  Affect | 21.6 ± 4.2 | 21.7 ± 3.3 | 22.4 ± 4.2 | 21.0 ± 4.5 | 19.9 ± 4.4 | 20.7 ± 3.5 | 22.1 ± 4.3 | 22.5 ± 4.6 | 23.2 ± 4.0 | | 20.3 ± 5.4 | 21.5 ± 4.0 | 21.5 ± 4.7 | b,d |
| Low  Activation | 16.4 ± 3.3 | 16.6 ± 3.3 | 16.9 ± 3.2 | 16.8 ± 3.7 | 16.6 ± 4.2 | 16.8 ± 3.0 | 15.8 ± 3.7 | 15.8 ± 2.3 | 15.9 ± 3.1 | | 15.3 ± 2.9 | 16.0 ± 2.9 | 15.6 ± 3.0 |  |
| Unactivated Unpleasant Affect | 9.1 ± 3.3 | 9.9 ± 3.6 | 10.3 ± 4.6 | 9.7 ± 3.2 | 10.5 ± 4.0 | 11.0 ± 3.8 | 7.9 ± 2.2 | 8.5 ± 2.4 | 8.5 ± 3.0 | | 9.4 ± 3.1 | 9.5 ± 3.4 | 9.2 ± 2.5 | b,f |
| Activated Unpleasant Affect | 7.6 ± 0.5 | 8.0 ± 2.4 | 8.8 ± 3.5 | 8.5 ± 3.2 | 9.0 ± 2.7 | 9.1 ± 3.1 | 7.6 ± 2.0 | 7.6 ± 1.9 | 7.4 ± 1.6 | | 9.0 ± 2.6 | 8.1 ± 2.5 | 8.1 ± 2.6 | b,f |
| Participants consumed either the treatment [calcium β-hydroxy-β-methylbutyrate (HMB, 1.5 g) + Vitamin D_3_ (D,1000 IU)] or a placebo (calcium lactate) twice daily for 12 months. Circumplex model questionnaire was completed at 0, 3, 6, 9, and 12 months of treatment; data from 0, 6, and 12 months are presented. Data are presented as Mean ± SD. | | | | | | | | | | | | | | |
| Significant effects based on probabilities from SAS Proc Mixed repeated measure model of analysis. The model included starting value, gender, treatment, exercise, time, treatment by exercise interaction, treatment by time interaction, exercise by time interaction, and treatment by exercise by time interaction. Only those participants completing 12 months of study were included in the model.  ^a^treatment main effect, ^b^exercise main effect, ^c^time main effect, ^d^treatment by exercise interaction, ^e^treatment by time interaction, ^f^exercise by time interaction, ^g^treatment by exercise by time interaction (*p*<0.05 for all). | | | | | | | | | | | | | | |

| **Supplemental Table 10.** Effect of HMB+D with and without exercise on SF-36 health survey in older adults. | | | | | | | | | | | | | |
| --- | --- | --- | --- | --- | --- | --- | --- | --- | --- | --- | --- | --- | --- |
|  | No-Exercise | | | | | | Exercise | | | | | |  |
|  | HMB+D | | | Control | | | HMB+D | | | Control | | | Statistics |
|  | Month | | | Month | | | Month | | | Month | | |  |
|  | 0 | 6 | 12 | 0 | 6 | 12 | 0 | 6 | 12 | 0 | 6 | 12 |  |
| Parameter | (n=  25-27) | (n=  27) | (n=  26-27) | (n=  25) | (n=  25-26) | (n=  25-26) | (n=  30) | (n=  29-30) | (n=  30) | (n=  33-34) | (n=  33-34) | (n=  33) |  |
| Physical Functioning | 79 ± 22 | 80 ± 20 | 84 ± 16 | 76 ± 80 | 83 ± 19 | 76 ± 23 | 92 ± 11 | 87 ± 24 | 91 ± 9 | 89 ± 15 | 93 ± 9 | 90 ± 16 | b |
| Physical Role | 81 ± 33 | 88 ± 28 | 83 ± 29 | 71 ± 41 | 83 ± 30 | 78 ± 33 | 94 ± 19 | 88 ± 27 | 86 ± 28 | 88 ± 28 | 90 ± 23 | 87 ± 31 |  |
| Bodily Pain | 68 ± 23 | 68 ± 22 | 72 ± 21 | 68 ± 24 | 70 ± 24 | 68 ± 21 | 80 ± 17 | 81 ± 16 | 79 ± 21 | 75 ± 17 | 77 ± 17 | 81 ± 10 |  |
| General Health | 77 ± 13 | 76 ± 14 | 77 ± 15 | 76 ± 11 | 76 ± 13 | 78 ± 12 | 81 ± 13 | 78 ± 16 | 81 ± 15 | 80 ± 14 | 85 ± 15 | 82 ± 13 |  |
| Vitality | 67 ± 12 | 66 ±14 | 67 ± 15 | 61 ± 18 | 61 ± 17 | 63 ± 19 | 70 ± 15 | 74 ± 18 | 74 ± 17 | 65 ± 15 | 68 ± 16 | 68 ± 19 | b,g |
| Social Functioning | 93 ± 10 | 88 ± 19 | 90 ± 15 | 89 ± 18 | 82 ± 23 | 85 ± 17 | 98 ± 5 | 95 ± 10 | 95 ± 13 | 90 ± 15 | 94 ± 11 | 93 ± 15 | b |
| Emotional Role | 91 ± 24 | 91 ± 18 | 96 ± 14 | 92 ± 22 | 82 ± 29 | 83 ± 30 | 97 ± 10 | 99 ± 6 | 86 ± 12 | 92 ± 19 | 94 ± 16 | 96 ± 14 | b |
| Participants consumed either the treatment [calcium β-hydroxy-β-methylbutyrate (HMB, 1.5 g) + Vitamin D_3_ (D,1000 IU)] or a placebo (calcium lactate) twice daily for 12 months. The SF-36 health survey was completed at 0, 3, 6, 9, and 12 months of treatment; data from 0, 6, and 12 months are presented. Data are presented as Mean ± SD. | | | | | | | | | | | | | |
| Significant effects based on probabilities from SAS Proc Mixed repeated measure model of analysis. The model included starting value, gender, treatment, exercise, time, treatment by exercise interaction, treatment by time interaction, exercise by time interaction, and treatment by exercise by time interaction. Only those participants completing 12 months of study were included in the model.  ^a^treatment main effect, ^b^exercise main effect, ^c^time main effect, ^d^treatment by exercise interaction, ^e^treatment by time interaction, ^f^exercise by time interaction, ^g^treatment by exercise by time interaction (*p*<0.05 for all). | | | | | | | | | | | | | |

|  |  | |  |  |  |  |  |  |  |  |  |  |  |  |
| --- | --- | --- | --- | --- | --- | --- | --- | --- | --- | --- | --- | --- | --- | --- |
| **Supplemental Table 11.** Modified intent-to-treat analysis of body composition changes based on DXA analysis after 6 and 12 months of supplementation with HMB+D or control with or without exercise. | | | | | | | | | | | | | |  |
|  | No Exercise | | | | | | | Exercise | | | | | |  |
|  | HMB+D | | | | Control | | | HMB+D | | | Control | | |  |
|  |  | Month | |  |  | Month |  |  | Month |  |  | Month |  | |
|  | 0 | 6 (Δ  from 0) | | 12 (Δ from 0) | 0 | 6 (Δ  from 0) | 12 (Δ from 0) | 0 | 6 (Δ  from 0) | 12 (Δ from 0) | 0 | 6 (Δ  from 0) | 12 (Δ from 0) | |
| Parameter | n=29 | n=29 | | n=29 | n=31 | n=31 | n=31 | n=33 | n=33 | n=33 | n=36 | n=36 | n=36 | |
| Lean mass, kg | 49.1 ± 2.1 | 0.45 ± 0.30 | | -0.08 ± 0.32 | 53.3 ± 1.6 | -0.05 ± 0.29 | 0.11 ± 0.31 | 50.9 ± 2.2 | 0.38 ± 0.28 | 0.20 ± 0.30 | 51.9 ± 1.9 | 0.37 ± 0.28 | 0.25 ± 0.29 | |
| Muscle mass,  kg | 46.6 ± 2.0 | 0.42 ± 0.30 | | -0.11 ± 0.32 | 50.6 ± 1.5 | -0.08 ± 0.29 | 0.06 ± 0.31 | 48.4 ± 2.1 | 0.37 ± 0.28 | 0.19 ± 0.30 | 49.4 ± 1.8 | 0.34 ± 0.27 | 0.23 ± 0.29 | |
| Fat mass, kg | 34.1 ± 1.7 | 0.08 ± 0.42^c^ | | 0.05 ± 0.52 | 37.8 ± 1.8 | 0.40 ± 0.41^c^ | 0.33 ± 0.52 | 33.2 ± 1.8 | -0.47 ± 0.39^c^ | -0.50 ± 0.49 | 31.8 ± 1.4 | -0.73 ± 0.38^c^ | -0.59 ± 0.48 | |
| Body fat, % | 40.9 ± 0.9 | -0.04 ± 0.31^c^ | | 0.15 ± 0.35^c^ | 41.3 ± 1.5 | 0.22 ± 0.31^c^ | 0.11 ± 0.37^c^ | 39.2 ± 1.1 | -0.49 ± 0.29^c^ | -0.45 ± 0.35^c^ | 38.0 ± 1.0 | -0.70 ± 0.28^c^ | -0.75 ± 0.34^c^ | |
| Trunk lean  mass, kg | 23.8 ± 1.1 | 0.26 ± 0.17 | | 0.05 ± 0.19 | 25.8 ± 0.7 | -0.02 ± 0.16 | 0.21 ± 0.18 | 24.6 ± 1.0 | 0.18 ± 0.15 | 0.11 ± 0.18 | 24.8 ± 0.9 | -0.06 ± 0.15 | 0.12 ± 0.17 | |
| Trunk fat  mass, kg | 17.2 ± 1.0 | 0.10 ± 0.26^c^ | | 0.04 ± 0.32 | 19.4 ± 0.9 | 0.31 ± 0.26^c^ | 0..17 ± 0.31 | 17.1 ± 1.0 | -0.30 ± 0.24^c^ | -0.30 ± 0.30 | 16.5 ± 0.9 | -0.57 ± 0.24^c^ | -0.31 ± 0.29 | |
| Trunk fat, % | 41.5 ± 1.0 | -0.05 ± 0.39^c^ | | 0.00 ± 0.43 | 42.6 ± 1.3 | 0.44 ± 0.39^c^ | 0.02 ± 0.46 | 40.5 ± 1.1 | -0.55 ± 0.37^c^ | -0.43 ± 0.43 | 39.4 ± 0.9 | -0.86 ± 0.36^c^ | -0.84 ± 0.42 | |
| Appendicular  fat mass, kg | 15.6 ± 0.8 | -0.03 ± 0.17 | | 0.03 ± 0.24 | 17.1 ± 1.1 | 0.11 ± 0.19 | 0.22 ± 0.24 | 14.6 ± 0.8 | -0.16 ± 0.17 | -0.17 ± 0.22 | 13.9 ± 0.7 | -0.14 ± 0.17 | -0.26 ± 0.22 | |
| Appendicular  lean mass, kg | 21.5 ± 1.0 | 0.28 ± 0.18 | | 0.16 ± 0.17 | 23.7 ± 0.8 | -0.23 ± 0.17 | -0.39± 0.17 | 22.5 ± 1.0 | 0.23 ± 0.16 | 0.16 ± 0.16 | 23.3 ± 1.0 | 0.47 ± 0.16 | 0.20 ± 0.16 | |
| Bone mineral  density, g/cm^3^ | 1.14 ± 0.02 | -0.02 ± 0.02 | | -0.02 ± 0.02 | 1.20 ± 0.03 | 0.01 ± 0.02 | 0.02 ± 0.02 | 1.13 ± 0.02 | -0.03 ± 0.02 | -0.03 ± 0.02 | 1.23 ± 0.09 | -0.05 ± 0.02 | -0.04 ± 0.02 | |
|  |  |  | |  |  |  |  |  |  |  |  |  |  | |

Participants consumed either the treatment [calcium β-hydroxy-β-methylbutyrate (HMB, 1.5 g) + Vitamin D_3_ (D,1000 IU)] or a placebo (calcium lactate) twice daily for 12 months. Data were analyzed by using a SAS Proc Mixed model of covariance analysis on the change at 3, 6, 9, and 12 months (6 and 12 months only for DXA). The model included the starting value as a covariate, sex, treatment, exercise, and treatment by exercise interaction. All participants completing 6 months of study were included in the model. Data are presented as Mean ± SE.

DXA, dual energy x-ray absorptiometry

a - treatment * exercise interaction (*p*< 0.05), b – main effect of treatment (*p*< 0.05), c – main effect of exercise (*p*< 0.05).
